# Vasculature in the mouse colon and spatial relationships with the enteric nervous system, glia, and immune cells

**DOI:** 10.3389/fnana.2023.1130169

**Published:** 2023-06-02

**Authors:** Lixin Wang, Pu-Qing Yuan, Yvette Taché

**Affiliations:** ^1^Department of Medicine, Vatche and Tamar Manoukian Division of Digestive Diseases, David Geffen School of Medicine, University of California, Los Angeles, Los Angeles, CA, United States; ^2^Veterans Affairs Greater Los Angeles Healthcare System, Los Angeles, CA, United States

**Keywords:** colon, immunohistochemistry, microvessel, mouse, vasculature, wheat germ agglutinin

## Abstract

The distribution, morphology, and innervation of vasculature in different mouse colonic segments and layers, as well as spatial relationships of the vasculature with the enteric plexuses, glia, and macrophages are far from being complete. The vessels in the adult mouse colon were stained by the cardiovascular perfusion of wheat germ agglutinin (WGA)-Alexa Fluor 448 and by CD31 immunoreactivity. Nerve fibers, enteric glia, and macrophages were immunostained in the WGA-perfused colon. The blood vessels entered from the mesentery to the submucosa and branched into the capillary networks in the mucosa and muscularis externa. The capillary net formed anastomosed rings at the orifices of mucosa crypts, and the capillary rings surrounded the crypts individually in the proximal colon and more than two crypts in the distal colon. Microvessels in the muscularis externa with myenteric plexus were less dense than in the mucosa and formed loops. In the circular smooth muscle layer, microvessels were distributed in the proximal, but not the distal colon. Capillaries did not enter the enteric ganglia. There were no significant differences in microvascular volume per tissue volume between the proximal and distal colon either in the mucosa or muscularis externa containing the myenteric plexus. PGP9.5-, tyrosine hydroxylase-, and calcitonin gene-related peptide (CGRP)-immunoreactive nerve fibers were distributed along the vessels in the submucosa. In the mucosa, PGP9.5-, CGRP-, and vasoactive intestinal peptide (VIP)-immunoreactive nerves terminated close to the capillary rings, while cells and processes labeled by S100B and glial fibrillary acidic protein were distributed mainly in the lamina propria and lower portion of the mucosa. Dense Iba1 immunoreactive macrophages were closely adjacent to the mucosal capillary rings. There were a few macrophages, but no glia in apposition to microvessels in the submucosa and muscularis externa. In conclusion, in the mouse colon, (1) the differences in vasculature between the proximal and distal colon were associated with the morphology, but not the microvascular amount per tissue volume in the mucosa and muscle layers; (2) the colonic mucosa contained significantly more microvessels than the muscularis externa; and (3) there were more CGRP and VIP nerve fibers found close to microvessels in the mucosa and submucosa than in the muscle layers.

## Introduction

Morphology supports the notion that “proximity defines functionality” (Mischopoulou et al., 2022). The blood supplies to the visceral organs play a major role in physiological functions and pathology as they contribute to the transport of oxygen, nutrients, immune signals, hormones, and therapeutic treatments. The visceral vascular structures are organ-specific and associated with their functions. For instance, the renal glomeruli and arterioles control both in- and out-flow (Hill et al., 2003; Murray and Paolini, 2022), and the “portal triad” in the liver and the hepatic sinusoids along the hepatocytes in the lobules return blood from the digestive tract and spleen to the liver (Gibert-Ramos et al., 2021). Compared to other visceral organs (Cao et al., 2018; Mühlfeld, 2021; Luxán and Dimmeler, 2022; Zhu et al., 2022), including the stomach and small intestine (Fu et al., 2013; Jaffey et al., 2021), the colonic vasculature is less thoroughly imaged in the different segments and layers (Ravnic et al., 2005; Ma et al., 2007; Porvasnik et al., 2010). The colon is an organ involved in fluid and electrolyte homeostasis, microbiota–gut–brain communication, and the expulsion of body waste. It is recognized that the formation and distribution of vascular structures are important for the communications between systemic humoral and local signals associated with the various functions of the colon. Moreover, alterations of blood vasculature were found in colonic pathologies, such as inflammatory bowel disease (de Fontgalland et al., 2014), cancer (Chang et al., 2000; den Uil et al., 2019; Kampoli et al., 2022), and aging (Boley et al., 1977; Korotinski et al., 2005; Ungvari et al., 2018; Alves et al., 2020).

The autonomic nerves are involved in modulating the gut blood flow (Király et al., 1998; Pénicaud, 2017). The blood vessels in the gastrointestinal tract are innervated by both extrinsic and intrinsic nerves containing neurotransmitters and neuropeptides from the sympathetic, sensory afferents, and neurons in the enteric ganglia (De Fontgalland et al., 2008; Li et al., 2008; Fu et al., 2013). The importance of extrinsic and intrinsic innervation of blood vessels in colonic functions has been well-delineated (Smith and Koh, 2017; Fung and Vanden Berghe, 2020). However, whether the enteric glia play a major role in regulating local blood flow is little known (Seguella and Gulbransen, 2021). In particular, the anatomical profiling of colonic vasculature is still not yet comprehensive to support whether there are interactions between enteric glia and immune cells with the blood vessels (Schneider et al., 2019; Seguella and Gulbransen, 2021). This knowledge is fundamental for understanding their role in gut inflammation (Rosenberg and Rao, 2021; Seguella and Gulbransen, 2021) and neurodegenerative diseases, such as Parkinson's disease (Clairembault et al., 2015; Benvenuti et al., 2020).

Detailed morphology and distribution patterns of the colonic mucosal microcirculation in rodents or humans were described in early studies by a scanning electron microscope using vascular corrosion casts that removed the tissues (Browning and Gannon, 1986; Sun et al., 1992; Skinner and O'Brien, 1996; Ravnic et al., 2007). However, the relationship between microvessels and surrounding tissues could not be visualized. The method named “fluorescent vessel painting” labels the blood vessels by perfusion of a tracer into the vascular system. This method facilitates the visualization of vascular structures and surrounding cells labeled by immunohistochemistry. The tracers can bind to the endothelium, such as lipophilic dyes or lectins conjugated with fluorophores (Ravnic et al., 2005; Fu et al., 2013; Cao et al., 2018). Wheat germ agglutinin (WGA) is a plant-derived lectin, which conjugated with Alexa Fluor (AF) 448 and was used to label the blood vessels in the mouse jejunum (Fu et al., 2013). In addition, cluster of differentiation (CD) 31 (also known as platelet endothelial cell adhesion molecule, PECAM-1) is found on endothelial cells and elements of the innate immune system, including macrophages and lymphocytes. CD31 antibody can be used as an immunochemical marker of the vascular endothelium (Müller et al., 2002; Ma et al., 2007). Recent technological advances in tissue clearing, microscopy, and software for image processing also greatly facilitate the visualization of vascular and neural networks in three-dimensional (3D) structures and quantitative analysis of digitized images of organs and tissues, such as the brain or adipose tissues (Cao et al., 2018; Bennett and Kim, 2022).

The objectives of the present study were to demonstrate 3D microscopic images of vasculature in the different segments and layers of the mouse colon, and the proximity of vascular structures to neurons, glia, and macrophages. The vessels were labeled by WGA painting with/without immunofluorescence of CD31 combined with the following neuronal markers: PGP9.5 (pan-neuronal), tyrosine hydroxylase (TH, extrinsic sympathetic), vasoactive intestinal peptide (VIP, intrinsic secretomotor), and calcitonin gene-related peptide (CGRP, sensory) (De Fontgalland et al., 2008; Fu et al., 2013). To label glia, which are considered as components in the gut-vascular barrier (Scalise et al., 2021), we used the glial cell markers glial fibrillary acidic protein (GFAP) and S100B, which is a glial-specific calcium-binding protein of the S-100 protein family; this Ca^+2^/Zn^+2^-binding protein specifically expressed by enteric glial cells of the gastrointestinal tract displays the morphological and functional equivalent of astrocytes in the central nervous system (Cirillo et al., 2011). The macrophages in the periphery are equivalent to microglia in the brain. The colon has numerous macrophages labeled by ionized calcium-binding adaptor molecule 1 (Iba1), a microglia/macrophage-specific marker (Wang et al., 2022), and their distributions related to microvessels were also studied.

## Materials and methods

### Animals

For this study, 8- to 12-week-old male C57BL/6J mice (29 in total) were purchased from Jackson Laboratories (000664, Sacramento, CA). They were maintained as two per cage under standard conditions. Animal care and experimental procedures followed institutional ethical guidelines and conformed to the requirements of federal regulations for animal research conduct. All procedures were approved by the Animal Research Committee at the Veterans Affairs Greater Los Angeles Healthcare System (animal protocol #07013-17).

### Vasculature painting

WGA AF-488 (W11261, ThermoFisher Scientific) was diluted in 0.1 M phosphate-buffered saline (PBS) at 30 μg/ml. Mice were deeply anesthetized with 5% isoflurane. The blood system was flushed with 0.9% saline *via* a cardiac cannula, and then WGA-AF 488 (30 μg/g of body weight) was perfused at 1 ml/g of mouse body weight and a rate of 3 ml/min. In addition, four mice were perfused with 4% paraformaldehyde (P6148, Millipore-Sigma) in 0.1 M phosphate buffer (pH 7.4) following WGA perfusion.

### Tissue collection and preparation

After the cardiovascular perfusion of WGA, the whole colon was removed from the ileocecal junction to the end of the distal colon at the level of the pelvic brim where the iliac artery runs. The colon was flat-pinned on a Sylgard™ 184 silicone elastomer (Electron Microscopy Science, Hatfield, PA), fixed in 4% paraformaldehyde in 0.1 M phosphate buffer (pH 7.4) overnight at 4°C. Thereafter, colon samples were divided into the proximal, mid, and distal colon (Wang et al., 2022). The mucosa of the proximal colon has unique structures, for example, the mucosal folds are folded toward the lumen and aligned to each other obliquely (Nava et al., 2011; Hugenholtz and de Vos, 2018; Wang et al., 2022). Thus, the mid colon starts at the location where the mucosal folds of the proximal colon disappeared. There is no clear line to define the mid and the distal colon. The mid colon wall is thicker and ~1.5 cm long. For 3D imaging, the colon with the whole thickness was cleared using the CLARITY protocol with hydrogel containing 4% acrylamide without paraformaldehyde (A4P0). The methodological details are as described in a previous report (Yang et al., 2014) and protocols (Yuan et al., 2021; dx.doi.org/10.17504/protocols.io.bqi2muge). Because the layers of the proximal colon with mucosal folds were poorly immunolabeled, the proximal colon samples were prepared using two additional procedures: all layers without the mucosa and separating the wall in two parts at the submucosal layer. The colon of mice perfused with the paraformaldehyde was postfixed overnight at 4°C and immersed in 20% sucrose for 2 days for cryoprotection. The proximal, mid, and distal colon were divided to 0.5–1 cm in length, embedded in Tissue-Plus^TM^ O.C.T. Compound (23-730-571, Fisher Scientific), snap-frozen, and stored in the deep freezer until sectioning for immunohistochemistry. Frozen colon samples of all segments were transversely sectioned at 200 μm in a cryostat to visualize the colon wall layers. Depending on the need for the immunostaining, the flat mounts were trimmed into four to six pieces and transverse sections of the colon wall were collected in five to eight sets from each proximal, mid, and distal colon.

### Immunohistochemistry

Each immunolabeling was performed in three to six samples of each colonic segment obtained from different sample preparations (flat whole wall, mucosa removed, colon wall separated in the submucosal layer, and sections). The total sample numbers from each colonic segment for each immunomarker were 3–19.

The flat mounts and transverse colonic sections of mice perfused with WGA-AF 488 were incubated according to the following steps: (1) 2% Triton-X 100 and 10% normal donkey serum in 0.01 M PBS for 1 h at room temperature (RT) and then overnight at 4°C; (2) primary antibodies (Table 1) in 0.3% Triton-X 100-PBS for 2 h RT, followed by 2–5 days at 4°C; and (3) secondary antibodies (donkey anti-rat IgG Alexa Fluor 594 for CD31 and donkey anti-rabbit Alexa Fluor 555 for other primary antibodies) for 3 h at RT, then overnight at 4°C. After immunolabeling, the colonic tissues were mounted onto a glass in a frame (iSpacer, Sunjin Lab, Hsinchu City, Taiwan, R.O.C.) (Yuan et al., 2021), and then sealed by glass coverslip in Vectashield anti-fade mounting medium (Vector Laboratories, Burlingame, CA) or RIMS (refractive index matching solution) (Yang et al., 2014).

**Table 1:** Primary antibodies.

**Antibody**	**RRID**	**Species**	**Source**	**Catalog No**.	**Dilution**
CD31	AB_393571	Rat	BD Biosciences	550274	1:50
PGP9.5	AB_10891773	Rabbit	Abcam	ab108986	1:1,000
αCGRP	AB_518147	Rabbit	Peninsula	T-4032	1:2,000
VIP	AB_2890602	Rabbit	CURE/UCLA	ab7913	1:1,000
TH	AB_390204	Rabbit	Millipore	AB152	1:1,000
GFAP	AB_305808	Rabbit	Abcam	ab7260	1:2,000
S100B	AB_882426	Rabbit	Abcam	AB56242	1:1,000
Iba1	AB_839504	Rabbit	WAKO	019-19741	1:1,000

RRID, Research Resource Identifiers.

#### Double immunolabeling

Proximal and distal colonic samples from 12 mice perfused with WGA-AF 488 perfused were double-immunostained for CD31 and other markers by mixing CD31 antibody with PGP9.5, CGRP, TH, or Iba1 antibodies (Table 1). The second antibodies were mixed with donkey anti-rat IgG AF 594 and donkey anti-rabbit IgG AF 647.

#### Counterstaining

DAPI (4′, 6-diamidino-2-phenylindole; 62247, ThermoFisher Scientific) 1:2,000, 30 min at RT.

### Imaging and quantitative analysis

Microscopic images were acquired with Z-stacks in Zeiss confocal microscopes (LSM 710 and 880, Carl Zeiss Microscopy GmbH, Germany), using objectives of 10X, 20X, and 63X. The optical sections were visualized at intervals of 2 or 2.5 μm in 10X, 1 μm in 20X, and 0.5 μm in 63X objectives. The image segmentation, quantitation, and visualization were performed using Imaris 9.8 and 9.9 for neuroscientists (Bitplane Inc., Concord, MA). The quantifications were performed in five to eight images for each layer in the proximal and distal colon. The volumes of microvessels were measured using the “Surface” module, calculated as a percentage in the tissue volume in 3D digital images, and compared between the proximal and distal colon.

### Statistical analysis

The analysis was performed using SigmaPlot 14 (Systat Software, Inc., San Jose, CA, USA). Data are presented as mean ± SEM. Comparisons of microvessel density between the proximal and distal colon were performed using Student's *t*-tests. *P*-values of < 0.05 were considered significant.

## Results

**Figure 1:**
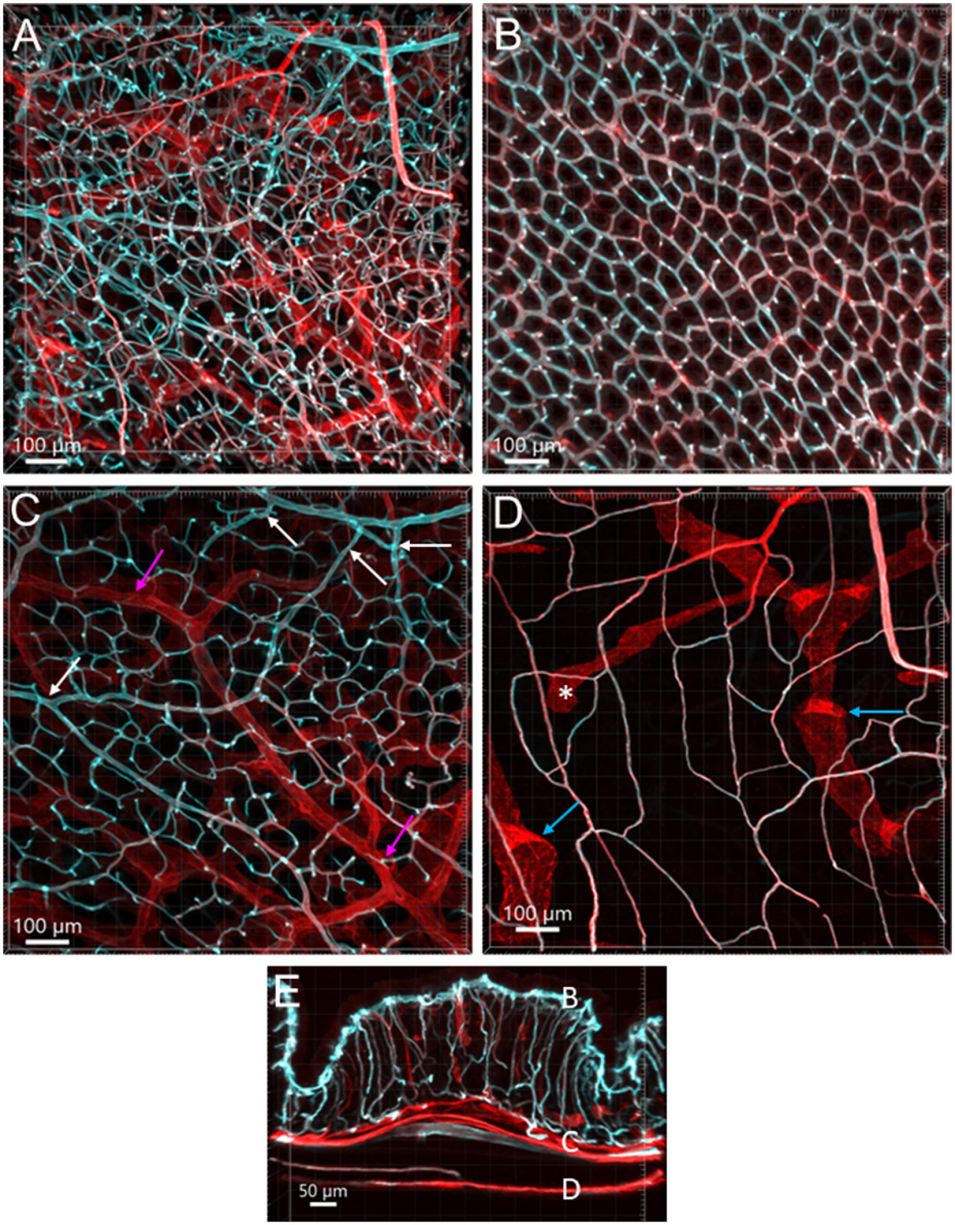
Vasculature of the mouse colon overviewed all layers from the serosa to the mucosa, labeled by WGA-AF 488 painting (cyan) and CD31 immunoreactivity (red) in an aboral segment of a mouse mid colon. Most of the microvessels were double-labeled (white). The vascular structures are similar in the mid and distal colon. **(A)** A 3D image of the flat wall of a mouse mid-colon with z-axis stacks. **(B–D)** Images are cropped from image **(A)** into three portions. **(B)** Capillary net in the mucosa near the lumen with the characteristic honeycomb-shaped rings connected together. **(C)** Vascular branches in the submucosal layer including the bottom of mucosal crypts. White arrows indicate the branches into the capillary nets at the bottom of mucosal crypts and magenta arrows indicate small veins. **(D)** Capillary net in the muscle layers near to the myenteric plexus. Blue arrows indicate the lymphatic vessels that had blind starting points (*), and wider and uneven diameters and valves (folds with brighter fluorescence). **(E)** A vertical section of the mid colon illustrates the location of layers cropped in images **(B–D)**. Scale bars are at the bottom of each image (same as in all figures).

**Figure 2:**
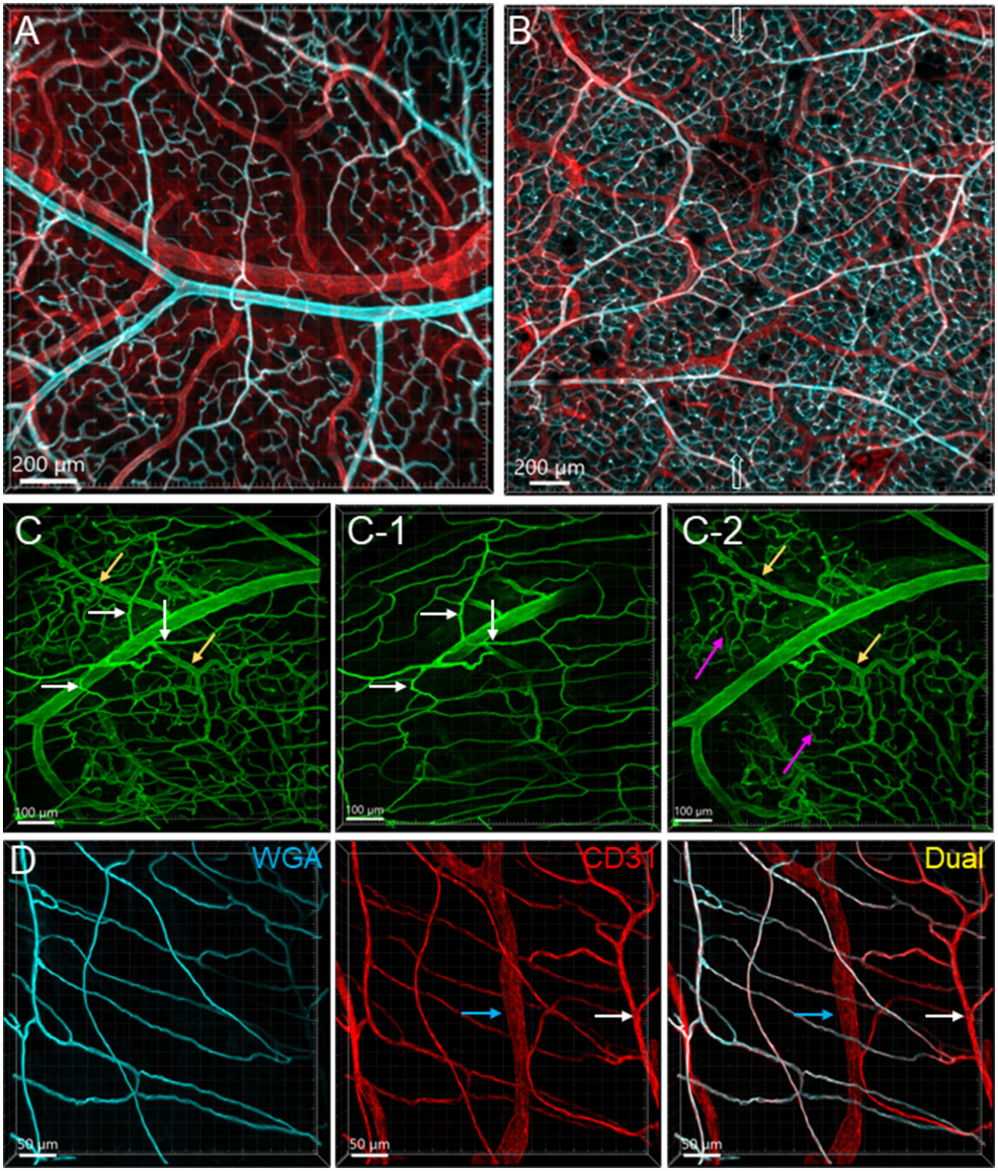
Vascular branches and connections in the mouse colon. The vessels were labeled by WGA painting (cyan in **A, B, D** and green in **C**) and CD31 immunoreactivity (red). **(A)** Submucosal vessels ramified and lead to capillary webs at the base of mucosal crypts. The cyan and white-colored vessels are the arterial branches till capillaries and red colored ones are veins. The sample was collected from the aboral segment of a mouse mid-colon. **(B)** Microphotograph shows that in the submucosa vessels from each side of the colon wall are connected, and the microvessels anastomosed in the networks near the antimesenteric margin (indicated by empty arrows) in a sample from the distal colon. **(C)** Microphotographs demonstrate that arterioles from the same artery lead into capillary networks in the mucosa (yellow arrows) and muscle layers (white arrows). The muscle layers in **(C-1)** and mucosa in **(C-2)** were cropped images from **(C)**. Magenta arrows indicate the capillary networks at the bottom of mucosal crypts. **(D)** Microvascular networks were labeled complementary by WGA and CD31 in muscle layers. White arrows indicate a CD31-labeled microvessel that was not perfused well by WGA. Blue arrows mark a lymphatic vessel labeled by CD31. The samples for images **(C, D)** were sampled from a mouse proximal colon.

**Figure 3:**
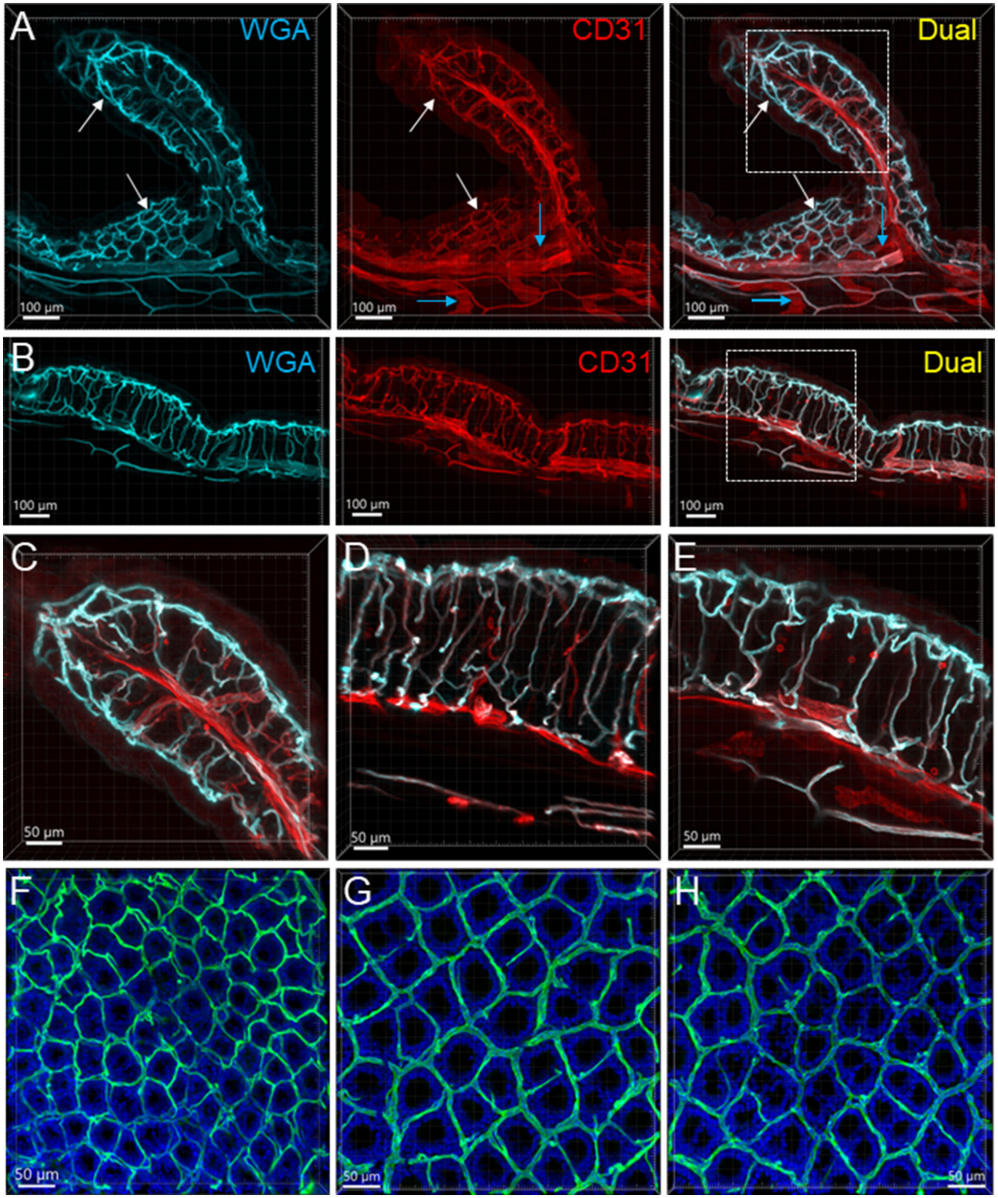
Mucosal microvessels in the mouse colon. **(A, B)** Transverse sections of a mouse proximal (Row **A**) and distal (Row **B**) colon. The vessels were painted by WGA perfusion (cyan) and CD31 immunoreactivity (red). The white arrows indicate the location of the capillary rings near the mucosal surface. Blue arrows: lymphatic vessels. **(C–E)** Magnification of microvessels in the proximal **(C)**, mid **(D)**, and distal colon **(E)**. The framed areas in the right panels of rows **(A, B)** are magnified in **(C, E)**, respectively. The capillaries connecting the capillary networks at the top and bottom of mucosal crypts were straighter in the mid and distal colon than in the proximal colon **(A–E)**. **(F–H)** WGA (green)-painted capillary rings in the mucosa of the proximal colon **(F)**, mid colon **(G)**, and distal colon **(H)**. Blue fluorescence is DAPI counterstaining. In the proximal colon, the capillary ring surrounded each mucosal crypt individually, while two crypts (see the DAPI-stained crypts) were in the distal colon. The proximal colon had more capillary rings, although smaller than the mid and distal colon in an area of the same size.

**Figure 4:**
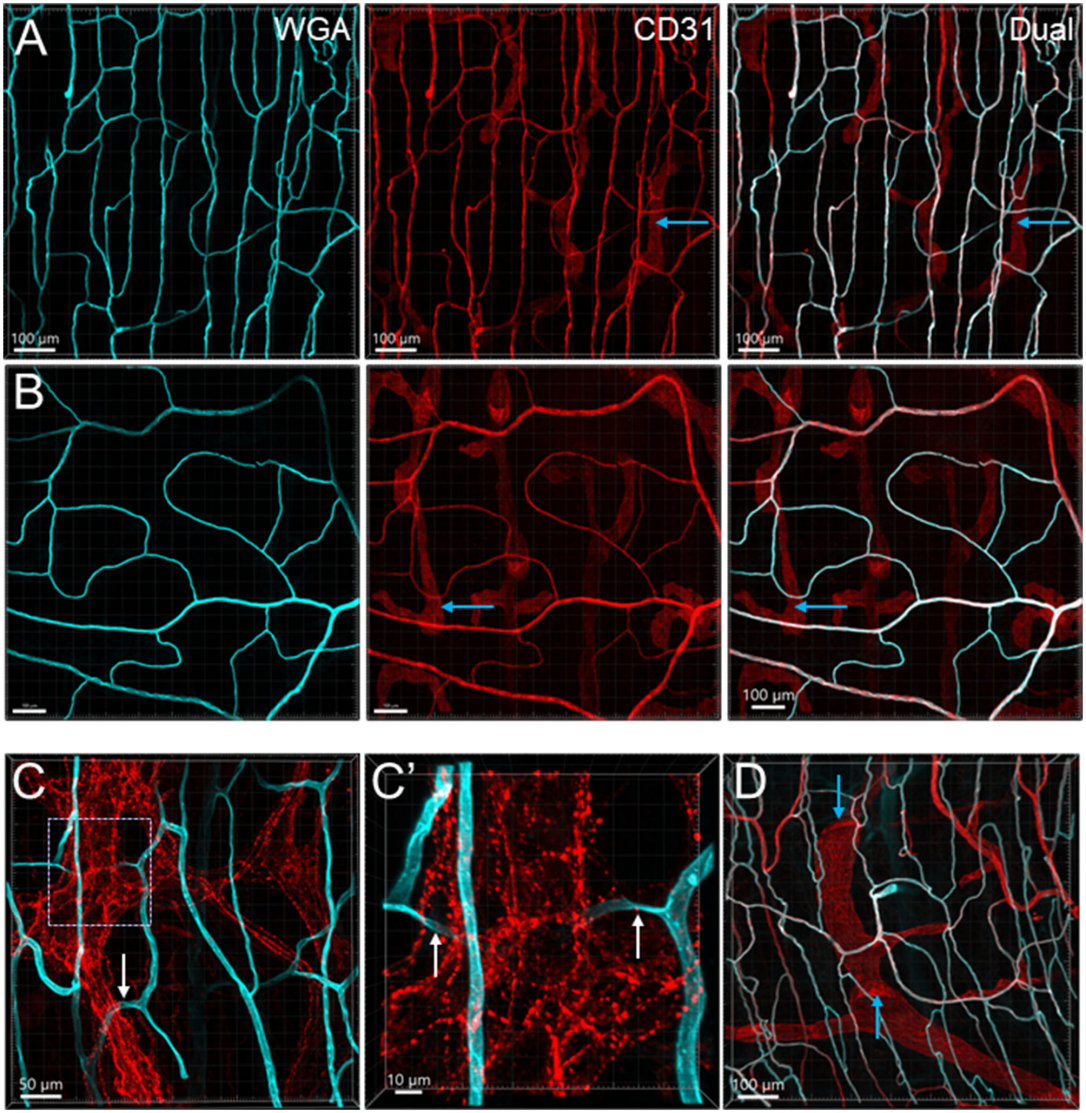
Microvessels in the muscle layers near the myenteric plexus in the mouse colon. Microvessels labeled by WGA perfusion (cyan) and CD31 immunoreactivity (red) in the muscle layers of the proximal (Row **A**) and distal colon (Row **B**). **(C)** The capillaries net (WGA, cyan) near the myenteric plexus (TH immunofluorescence, red) of the proximal colon. The capillaries passed through ganglion and interganglionic strands (marquee and arrows) and were magnified in **(C')**. The arrows indicate the capillary passing through. **(D)** Lymphatic vessels labeled with CD31 immunofluorescence with valves (blue arrows) in the muscle layer under the serosa. Several lymphatic vessels can also be seen in the panels of CD31 labeling in rows **(A, B)** (examples marked by blue arrows).

Perfusion of WGA painted the arteries, arterioles, and capillaries, while the veins were shown with faint or no fluorescence in the colon (Figures 1B, C, 2A, 7). CD31 (*n* = 13) immunostained the capillaries and small veins, but not most of the arteries in the colon flat mounts (Figures 1, 2A). WGA and CD31 double-labeled the majority of microvessels (Figures 1B–D, 2D, 3A–C, 4); and CD31 also labeled the lymphatic vessels (Figures 1D, 4A, B, D).

### Distribution of blood vessels

From the mesentery margin, the arteries and veins entered into the submucosal layer and divided into smaller branches toward the antimesentery forming loops (Figures 2A, B), with a few branches under the serosa. Often, the same vessels branched out to both mucosa and muscularis externa (Figure 2C). In the submucosa near the bottom of crypts, arterioles branched into the capillary net around the crypts (Figures 2A–C). A capillary net surrounded the orifice of the mucosal crypts in the shape of a honeycomb with anastomosed rings continuously beneath the epithelium (Figures 1B, 3F–H). The microvessels connected the vessels in the submucosa near the bottom of the mucosal crypts to the capillary rings on the top of the mucosa in perpendicular angles (Figures 3A–E). Microvascular webs were distributed in the longitudinal muscles and circumferentially in the circular muscles (Figure 4A). The capillaries occasionally traveled through spaces in the myenteric ganglia and interganglionic strands (Figure 4C).

**Figure 5:**
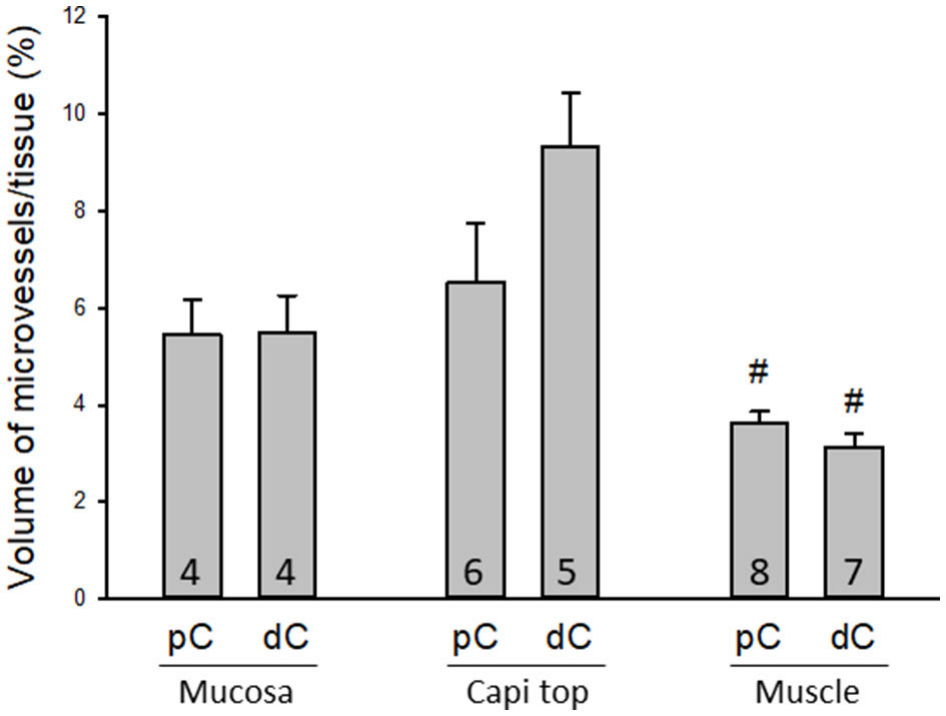
Quantifications of microvessels in the mouse colon. There were no significant differences between the proximal and distal colon when the microvessels were measured in 3D per tissue volume in three regions: mucosal microvessels in vertical sections (Mucosa), the top mucosal capillary rings (Capi top), and muscle layer near the myenteric plexus (Muscle). ^#^*p* < 0.05 mucosa vs. muscle layers in the proximal and distal colon, respectively. *N* = 4–8 mice in each group as indicated in each bar.

The proximal colon contained more vascular supplies than the distal colon (Figure 3). This is associated with the mucosal folds in the proximal colon, which increased mucosal volume. There are also many microvessels paralleled to the circular muscles in the proximal colon (Figure 4A), which were distributed up to the oral portions of mid colon contrasting with the distal colon where they were rarely found and are displayed simply as one layer of capillary loops (Figure 4B). The microvessels connecting the submucosal and the mucosal capillary rings beneath the epithelia were ramified more in the proximal colon than those in the mid and distal colon, which mostly went up straight (Figures 3A–E). Capillary rings individually surrounded the mucosal crypts in the proximal colon (Figure 3F), while in the distal colon, the capillary rings more frequently surrounded two (Figure 3H) or occasionally more crypts (data not shown). The proximal colon had more capillary rings, although smaller than those in the mid and distal colon in the same area in terms of size (Figures 3F–H). However, there were no significant differences between the proximal and distal colon when the microvascular densities were quantified in percent per tissue volume: top mucosal capillary rings (6.5 ± 1.2% vs. 9.3 ± 1.1%, respectively, *p* > 0.05, *n* = 6/group); nearby the myenteric plexus (3.6 ± 0.2% vs. 3.1 ± 0.3%, *p* > 0.05, *n* = 7–8/group); and mucosal microvessels in vertical sections (5.4 ± 0.7% vs. 5.5 ± 0.7%, *p* > 0.05, *n* = 4/group). The differences were significant when the data were compared between mucosa and muscle layers in the proximal and distal colon, respectively (Figure 5; *p* < 0.05).

Lymphatic vessels labeled by CD31 were located under the serosa and in the submucosa and lamina propria (Figures 1D, 3A, 4A, B, D, 7). They featured enlarged and uneven diameters, blind ends, and valves (Figures 1D, 4A, B, D).

### Nerve fibers and vessels

**Figure 6:**
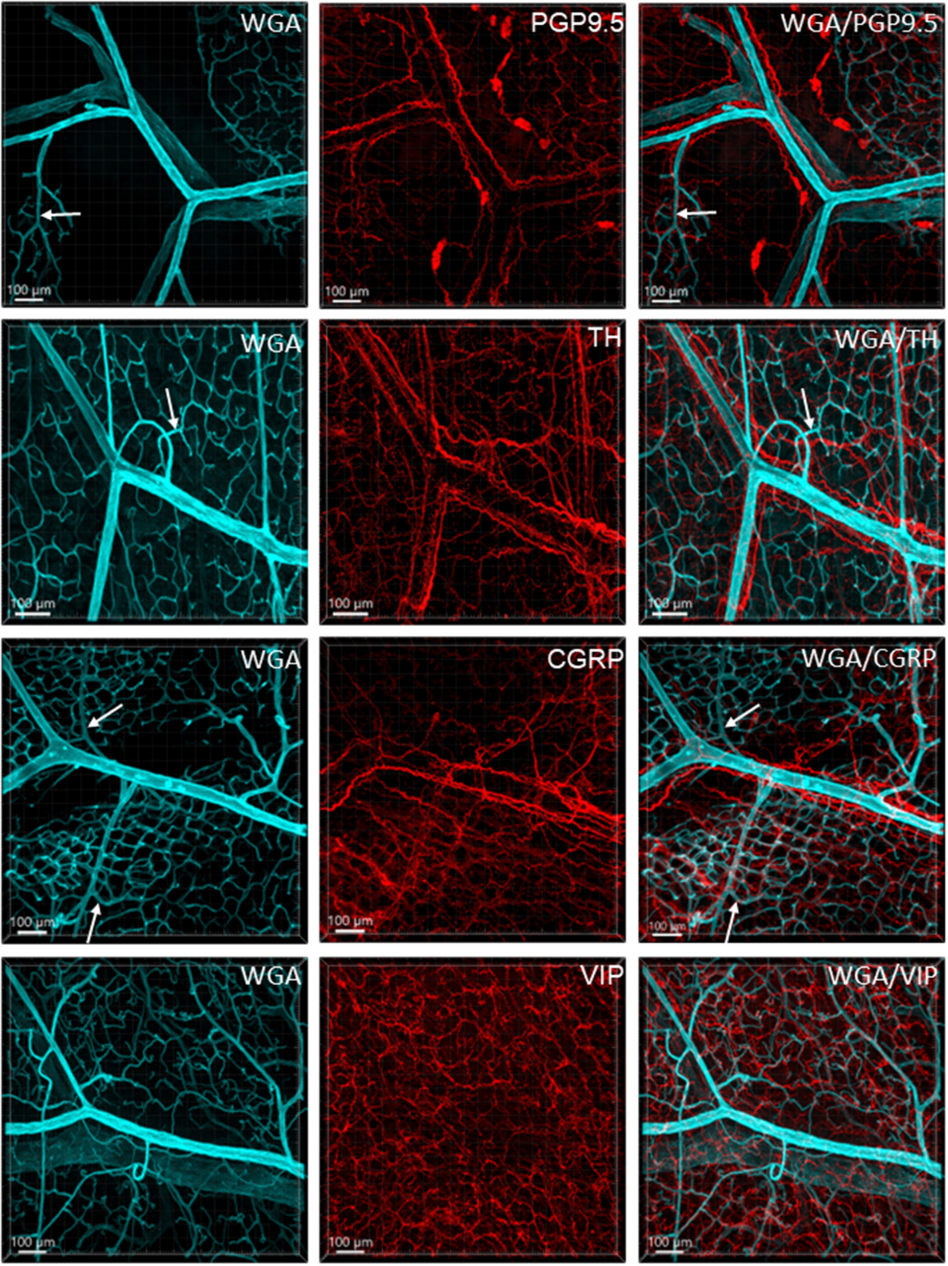
Immunoreactive nerve fibers and vascular branches in the submucosa of mouse colon. The vessels were painted by WGA perfusion (cyan), and for the nerve-vessel relationship, WGA perfused samples were immunolabeled in red for PGP9.5, TH, CGRP, and VIP (red). The nerve fibers were not found in the arterioles entering capillary webs (examples are indicated by arrows). The pattern is similar in different colonic segments. The samples were collected from the distal colon.

**Figure 7:**
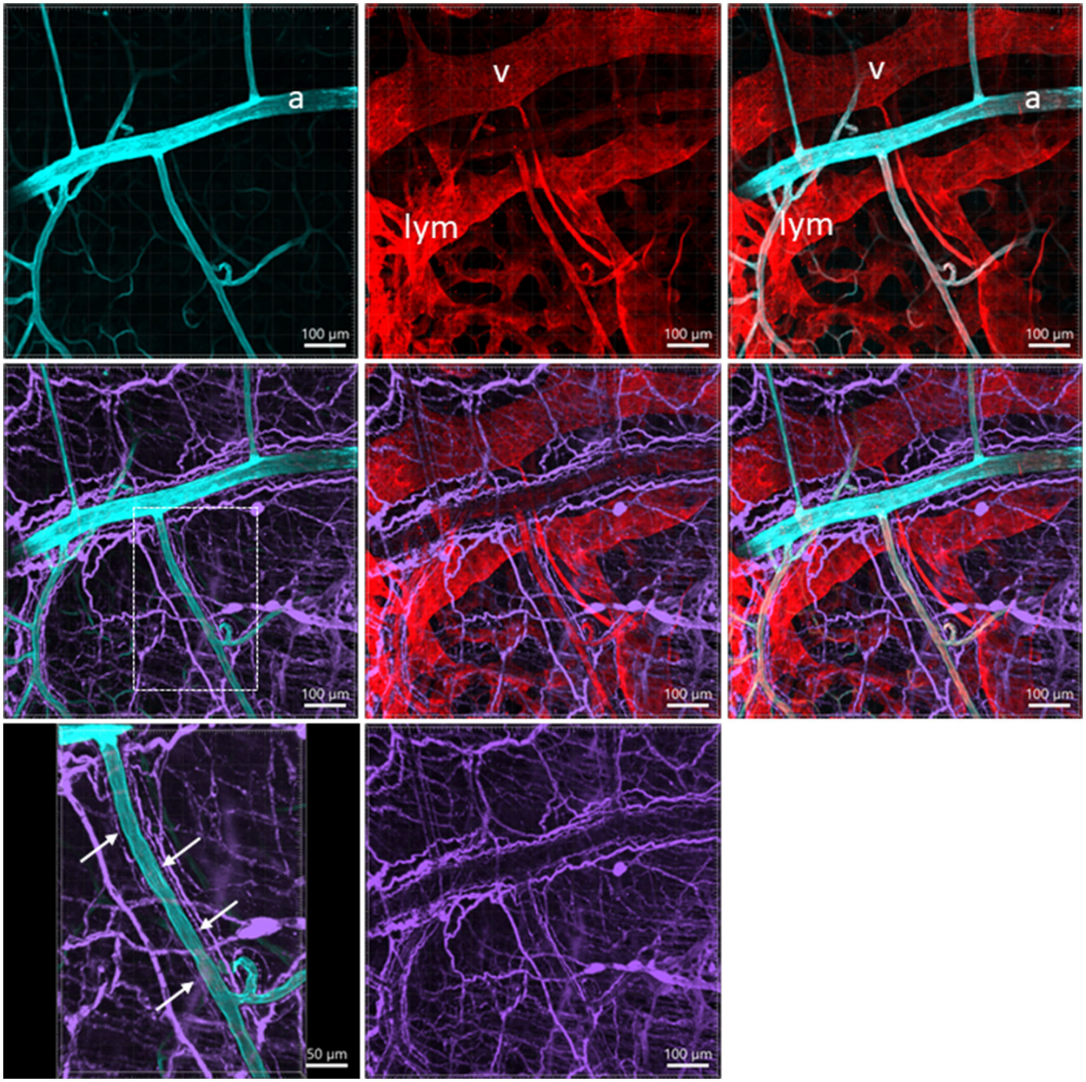
A photomicrograph of triple fluorescent labeling of vessels and nerves in the submucosa of a mouse mid-colon shown in different panels with various fluorescent labeling. The vessels were painted by WGA perfusion (cyan) combined with CD31 immunolabeling (red), and nerves by PGP9.5 immunostaining (purple). The arteries (a), veins (v), and lymphatic vessels (lym) accompanied each other. WGA perfusion did not stain the veins. Nerve fibers traveled along the arteries. The lower-left panel is enlarged in the area framed in the panel earlier, demonstrating the nerve fibers traveling along the blood vessels. The single fine nerve fibers adjacent to the vascular wall (arrows) were possibly those innervating the vessels.

**Figure 8:**
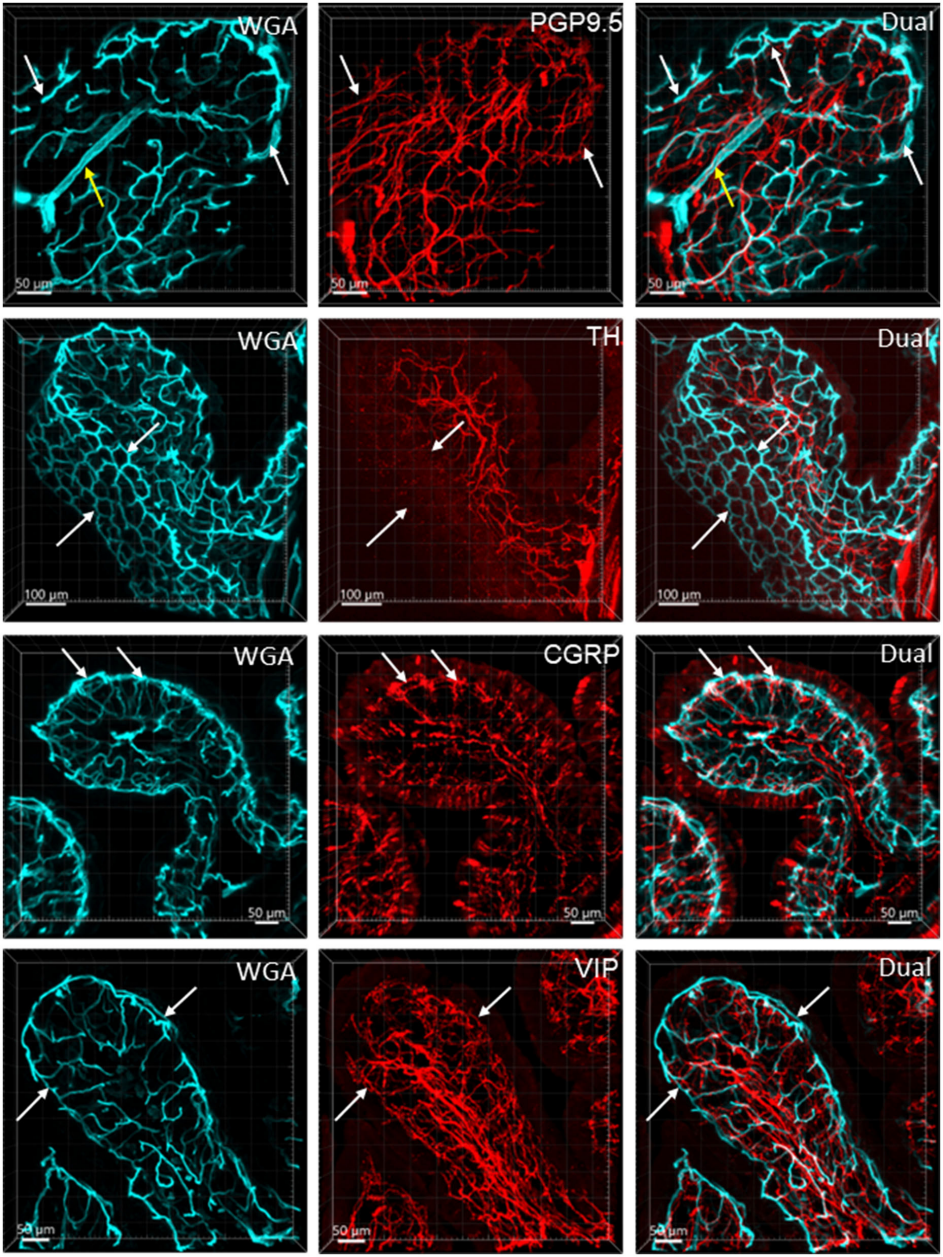
Nerve fibers and microvessels in the mucosa are shown in vertical sections of the mouse proximal colon. The vessels were painted by WGA perfusion (cyan) and PGP9.5, TH, CGRP, and VIP-ir nerve fibers were immunostained (red). More CGRP and VIP-ir than TH-ir nerve fibers are projected to the top of the mucosa (arrows). TH-ir terminals rarely reached the capillary rings at the mucosa top (arrows in the 2nd row). Yellow arrows in the top row indicate an arteriole in the lamina propria.

**Figure 9:**
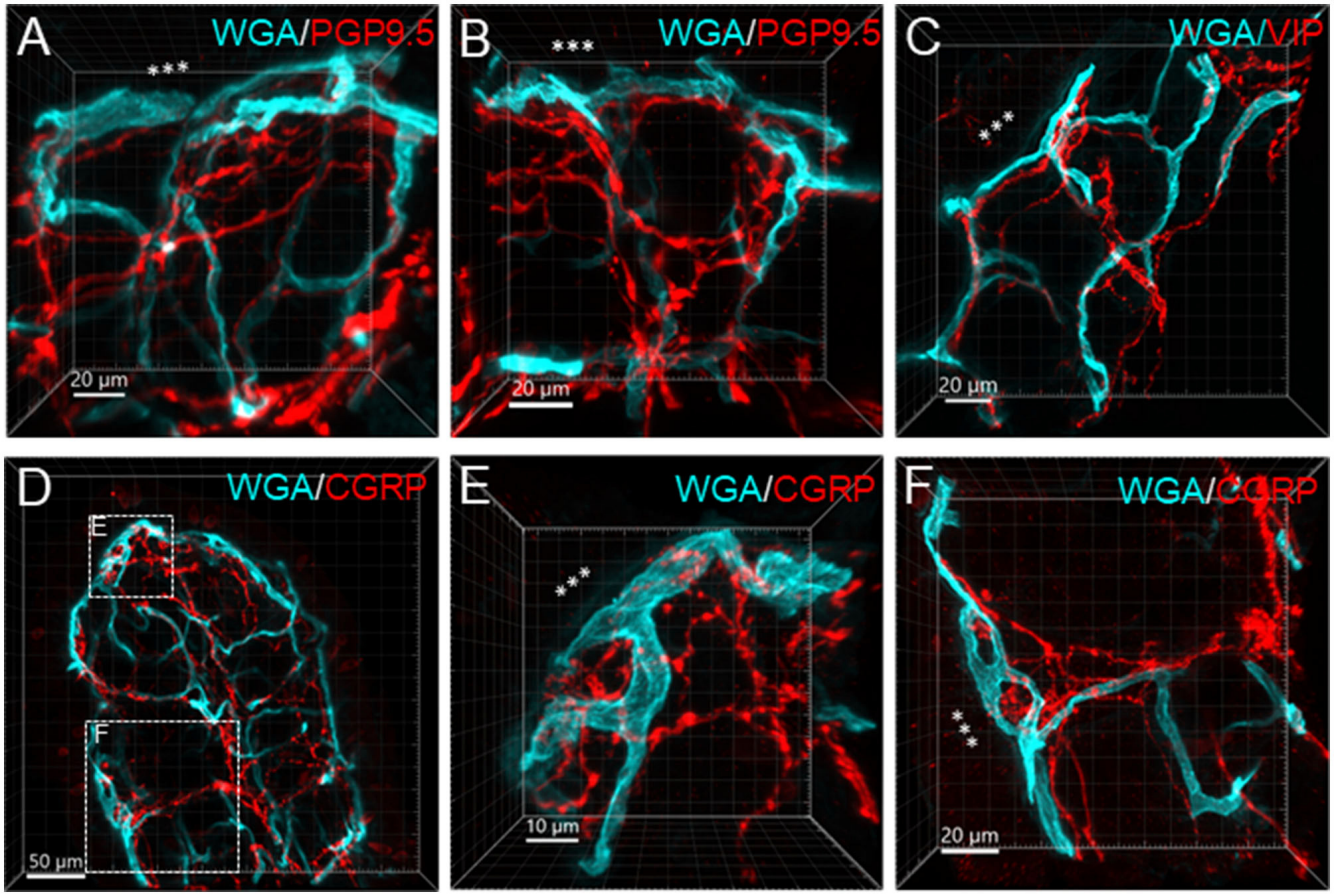
Examples illustrating nerve terminals distributed closely to the capillaries in the mucosa. Samples were from the proximal colon. Microphotographs at high magnification were acquired under 63X objective. **(A–D)** The vessels were painted by WGA perfusion (green), and nerve fibers were immunolabeled by PGP9.5, CGRP and VIP (red). **(E, F)** Show magnified areas framed in **(D)**. The “***” indicated the lumen site.

**Figure 10:**
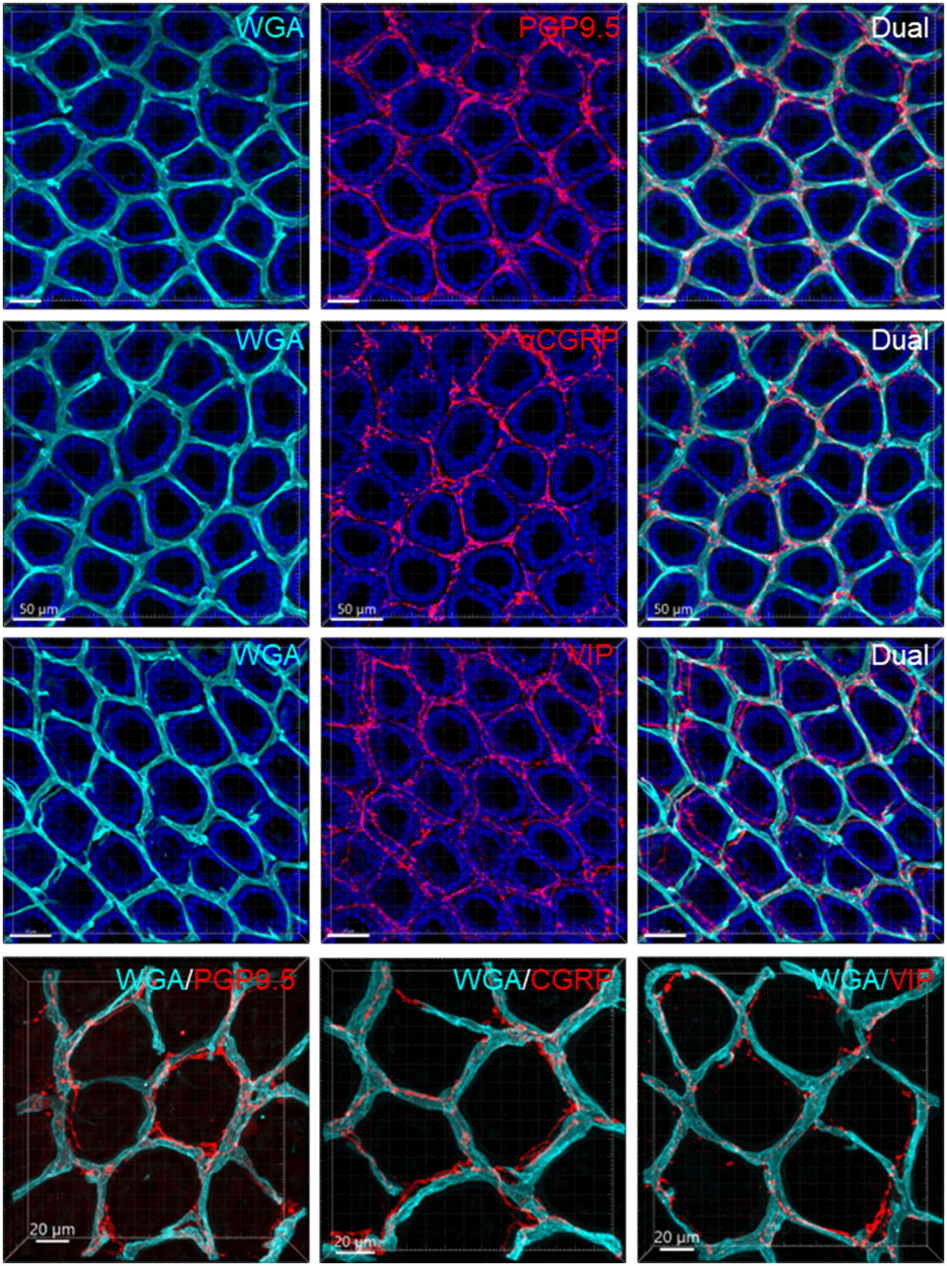
Capillary rings and nerve fibers at the top of the mucosa. The samples were from the mid-colon. The vessels were painted by WGA perfusion (cyan), nerve fibers were immunolabeled by PGP9.5, CGRP, and VIP (red), and the tissues were counterstained by DAPI (blue). The different fluorescent channels merged in the same image demonstrate the mucosa capillary net in relation to immunofluorescent nerve terminals. High magnifications (objective 63X) of double labeling of WGA with each immunostaining are in the bottom panels.

Figure 6 illustrates the nerve fibers in the submucosal layer labeled by PGP9.5 (*n* = 16), TH (*n* = 19), and CGRP (*n* = 15), traveling in small bundles along the arteries and veins, while VIP-immunoreactive (ir) nerve fibers (*n* = 13) were not found along the vascular branches. Single thin nerve fibers adjacent to the vascular wall possibly innervated the vessels (Figure 7). In the mucosa, a few PGP9.5, CGRP, and VIP nerve fibers projected up close to the top capillary rings (Figures 8–10). Neuronal cell bodies and processes adjacent to vasculature were rarely observed in the submucosal and myenteric plexuses or the muscularis externa (Figure 4C). The pattern was similar in the different colonic segments.

### Glia and macrophages

**Figure 11:**
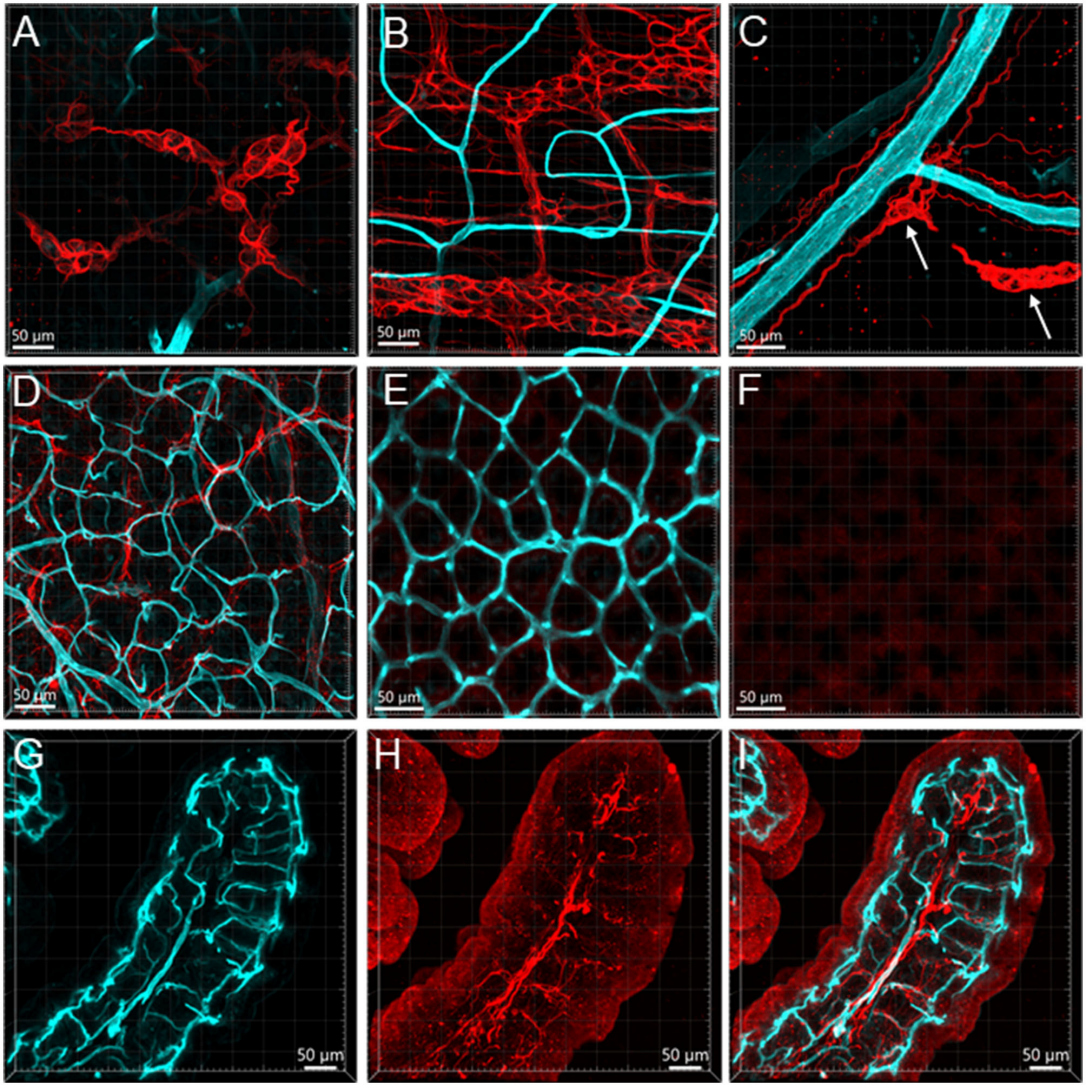
Vessels and GFAP-ir glia in the mouse colon. The vessels were painted by WGA (cyan) and glial cells were immunostained by GFAP (red). Samples in **(A–F)** were from the mid-colon, and the sample in G–I was from the proximal colon. **(A, B)** GFAP-ir glia in the submucosal plexus **(A)** and myenteric plexus **(B)**. The capillaries did not enter either of the plexuses. **(C)** GFAP-ir fibers surrounded vessels in the submucosa. **(D–F)** WGA-GFAP double labeling in the mucosa. **(D)** At the bottom of mucosal crypts; **(E)** capillary rings (WGA) near the lumen, no GFAP labeling found. **(F)** the red channel shows GFAP negative. **(G–I)** Portion of a mucosal fold from the proximal colon with WGA-painted microvessels and GFAP immunoreactive glial cells and processes. GFAP glia were not distributed around microvessels.

**Figure 12:**
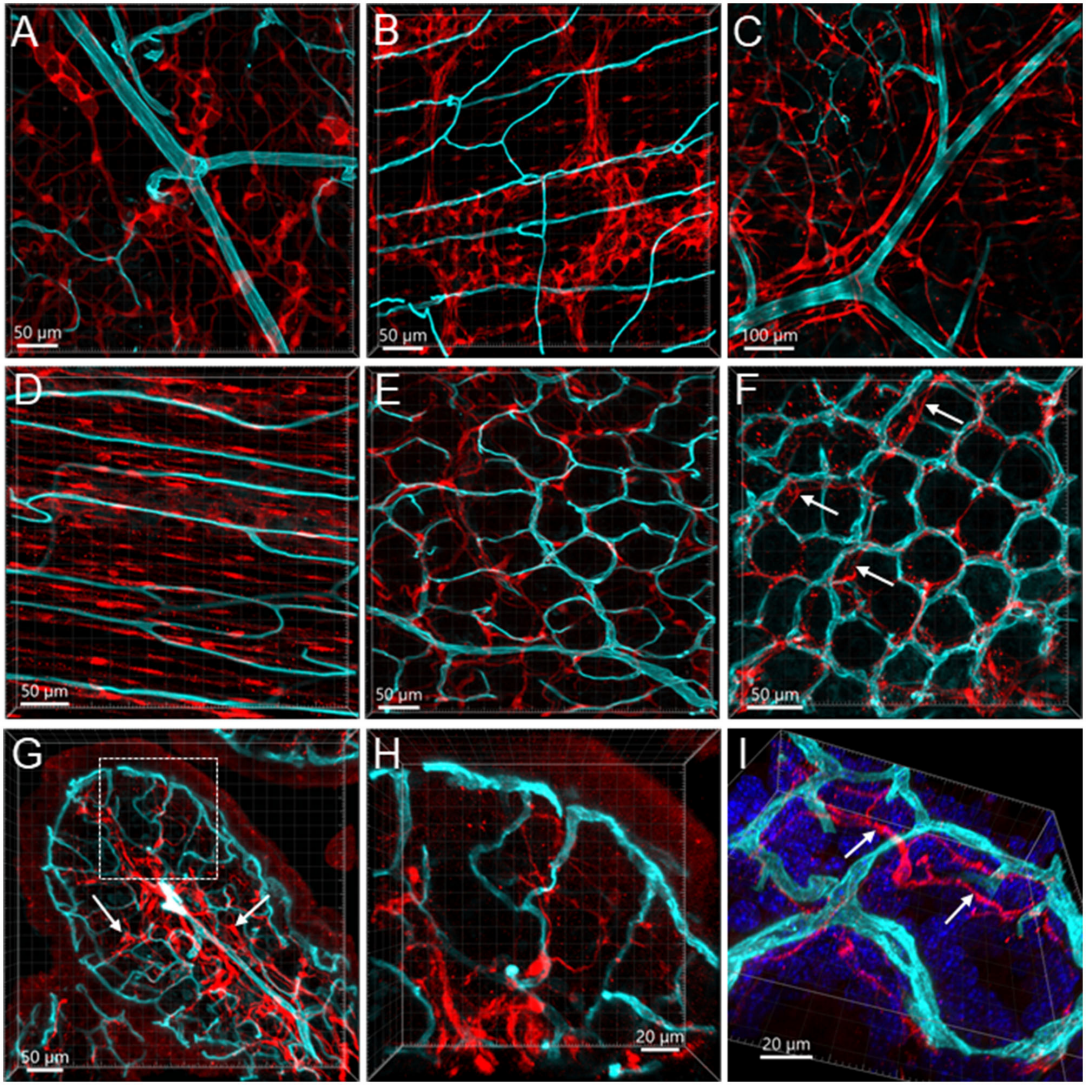
Vessels and S100B-ir cells in the mouse colon. The vessels were painted by WGA (cyan) and cells were immunostained by S100B (red). All the images are samples from the proximal colon. **(A, B)** S100B-ir cells in the submucosal plexus and myenteric plexus, respectively. The capillaries did not get into either of the plexuses. **(C)**: S100B-ir cell bodies and processes surrounded vessels in the submucosa. **(D)** S100B-ir cells and microvessels in the circular muscle layer. **(E)** In the submucosal layer, S100B-ir cells formed in a net close to the capillary net at the bottom of mucosal crypts. **(F)** S100B-ir cells located close to the top mucosal capillary network. Arrow indicates S100B labels under the capillary rings. **(G)** Transverse section of a mucosal fold in the proximal colon showing S100B-ir cells located densely in the lower portion of mucosa and lamina propria (arrows). **(H, I)** High magnifications demonstrate some S100B-ir processes at the top of mucosal crypts while not in apposition to the capillaries. The image in **(I)** is tilted to show the locations of capillaries and S100B-ir processes (arrows).

GFAP-ir (*n* = 3) glia cells were located in both submucosal and myenteric ganglia. The labeling was fibrous and did not show the glial cell bodies well (Figures 11A, B). Their processes reached the circular muscular layer but were not frequently in a close relationship with the vascular structures. Occasionally, GFAP fibers surrounded arteries and veins in the submucosa (Figure 11C), but they were not seen in the adjacent proximity of microvessels (Figures 11D–I). The GFAP-ir glial cells were mostly detected in the lamina propria and the lower portion of the mucosa. S100B (*n* = 10) labeled many cells in both the submucosal and myenteric plexuses. Compared to the GFAP-ir glia, the S100B-ir labeling was in the cytoplasm (Figures 12A, B). The S100B-ir cells were numerous in the muscle layers, submucosa, and mucosa (Figures 12C–F), while only a few of them approached in apposition to mucosal capillary networks (Figures 12G–I).

**Figure 13:**
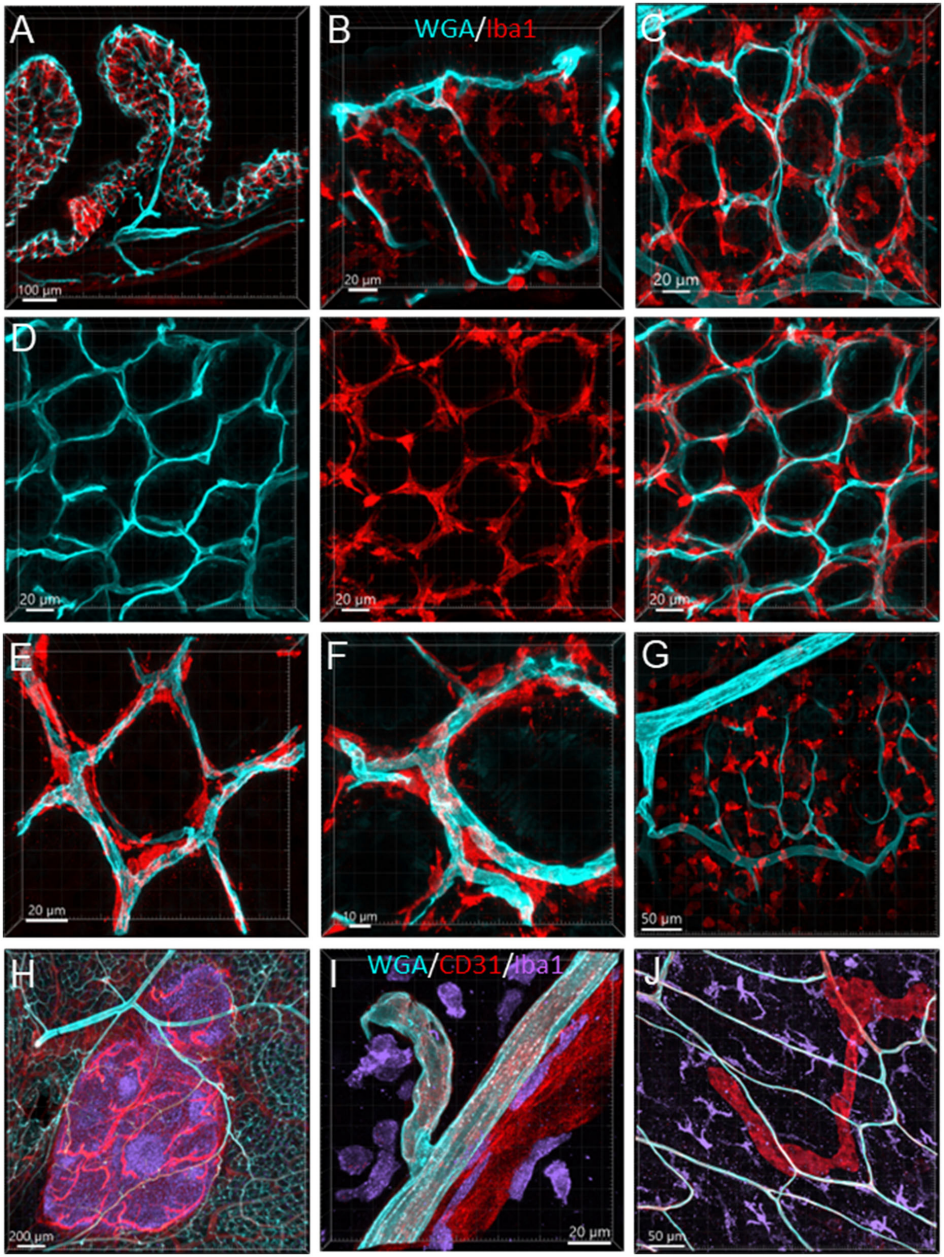
Vessels and macrophages in the mouse colon. **(A)** Iba1-ir macrophages (red) distributed in the mucosa shown in a vertical section of the proximal colon (microvessels painted by WGA, cyan). **(B)** Dense Iba1-ir macrophages at the top of mucosa at high magnification. **(C)** WGA-pained vessels and Iba1-ir macrophages (red) in the capillary net at the bottom of mucosal crypts. **(D)** Iba1-ir macrophages close to the capillary net at the top of mucosal crypts. The three panels displayed the same photomicrography in different channel(s). **(E)** High magnification of Iba1-ir macrophages in the capillary rings at the bottom of the mucosal crypt. **(F)** High magnification of Iba1-ir macrophages in the capillary rings at the mucosal crypts top. **(G)** Iba1-ir macrophages in the submucosa. **(H)** Vessels (WGA+CD31) in a Peyer's patch with many Iba1-ir cells inside (purple). **(I)** Iba1-ir cells (purple) near the vessels (WGA cyan+CD31 red) in the submucosa. **(J)** Iba1-ir macrophages (cyan) in the muscularis externa with WGA and CD31 dual-labeled capillaries and a CD31-ir lymphatic vessel (red).

The Iba1-ir macrophages (*n* = 4) were dense in the mucosa and distributed from the bottom to the top around the mucosal glands and adjacent to the capillaries (Figures 13A–F). Most of the macrophages were ramified, except some close to major vessels and in the submucosal layer (Figures 13G, I). In the muscularis externa, the Iba1-ir macrophages were shaped irregularly with the Iba1 immunostaining in fragmented cellular parts. They were spread randomly, and a few were in the proximity of the microvessels (Figure 13J).

The Peyer's patches contained dense Iba1-ir cells and vascular structures, i.e., arterioles, venules, capillaries, and lymphatic draining (Figure 13H) in all segments.

## Discussion

The vascular distribution patterns and morphology were different in the mouse colonic layers and segments. The microvessels densely surrounded the mucosa crypts in a honeycomb-like formation and were distributed circumferentially in the circular muscle layer and in larger loops near the myenteric plexus. The proximal colon contained more microvessels associated with the increased volume of mucosa in the mucosal folds and thicker circular muscle layer compared to the distal colon. The capillary networks in the mucosa and muscular layers between the proximal and distal colon varied morphologically, but not the microvascular volume per tissue volume. In the mucosa, there were CGRP-ir and VIP-ir nerve terminals and numerous macrophages labeled by Iba1. The capillaries were not distributed into the submucosal and myenteric nervous plexuses, and the GFAP and S100B-ir cells did not enclose microvessels in a sheath.

In the present study, WGA-AF 488 yielded a clean fluorescent vascular labeling in the mouse colon with high signal-to-noise contrast, although it was reported to have lower efficacy than another lectin, *Lycopersicon esculentum* agglutinin (Battistella et al., 2021). The fluorescent labeling was tolerant to the processing of tissue clearing and immunohistochemistry and did not have obvious quenching by laser scan. The perfusion of WGA did not fill all vessels, which could be related to the velocity of blood flow and dilation or contraction of arteries and arterioles. CD31 immunostained microvessels, but not all arteries and veins, possibly because these vessels were intact in the flat colonic mounts and limited the antibodies' penetration in the vascular wall. In thin sections, there was homogeneous strong staining of CD31 of the endothelia in all vessels, irrespective of vessel diameters (Müller et al., 2002). The different characteristics between WGA and CD31 labeling could be complementary to assess the vasculatures. In addition, CD31 labeled lymphatic vessels and most of them can be recognized by the morphological features, such as blind ends, enlarged uneven diameters, and valves with bright fluorescent labeling.

The mucosa structures are different in the small and large intestines. Instead of villi, the colon mucosa contains crypts, forming tube-like glands with openings to the lumen on the top. Accordingly, the formation of capillary beds is different from those in the mucosa of the small intestine of the mouse (Fu et al., 2013). Our study using WGA painting and CD31 immunolabeling depicted the capillary rings near the lumen in the mouse colonic mucosa consistent with those reported previously in mouse, rat, and human colon using vascular casting by polymerized compounds (Browning and Gannon, 1986; Sun et al., 1992; Skinner and O'Brien, 1996; Ravnic et al., 2007). It was described as “typically arranged in a regular, hexagonal honeycomb pattern around the mucosal glands”; and the pattern “is seen in all parts of the large intestine from the caecum to the rectum” in humans (Konerding et al., 2001). The advantage of WGA painting of the blood vessels over the vascular casting technique is that the tissues are intact, which allows study of the relationship between the vessels and surrounding structures.

In the brain, there is a correlation between neuronal and vascular density (Tsai et al., 2009), while in the gut, the vascular density is correlated with tissue specificity adapted to physiological demands (Fung and Vanden Berghe, 2020). In the mouse colon, the mucosa had denser capillary networks than the muscle layers. The mucosal capillary rings in the proximal colon surrounded the mucosal crypts individually and some areas had doubled layers, compared to two crypts and one layer in the distal colon. In addition, the mucosal folds increase the mucosal vascular volume in the proximal compared to the distal colon, although we did not detect a significant difference between the proximal and distal colon in the microvascular volume per tissue volume. In humans, the mucosal capillary networks were multi-layered in the ascending colon and almost single-layered in the sigmoid colon (Araki et al., 1996). In the ascending colon, there is a greater density, volume, and surface of mucosal microvessels (Skinner and O'Brien, 1996), which could be related to the prominent water absorption and electrolyte transport function taking place compared to the distal colon (Araki et al., 1996; Fung and Vanden Berghe, 2020).

A previous study using the same vessel painting method with WGA-AF 488 described that the capillaries make contact with the enteric ganglia in mouse jejunum (Fu et al., 2013); however, the contacts were demonstrated only in a few sparse locations. In the present study, microvessels in the structures of either the submucosal or myenteric plexus of the mouse colon were scarce. Capillaries traveled through tiny spaces in the ganglia and interganglionic strands sparsely and randomly, and there were no appositions when the 3D images were screened by optical sections at high magnifications. Supportive evidence using electron microscopy showed that capillaries do not enter the myenteric plexus in mouse jejunum (Gershon and Bursztajn, 1978) and that the myenteric ganglia in guinea pigs were “completely surrounded by a basal lamina and isolated from the connective tissues and blood vessels” (Gabella, 1972). In the rat distal colon, a basement membrane enclosed the submucosal ganglia and nerve strands, and the ganglia were free of blood vessels and connective tissue (Mestres et al., 1992). Morphologically, the enteric nervous plexuses are intrinsic networks consisting of tiny ganglia and interganglionic strands. Physiologically, the colonic movements, i.e., propagation and peristaltic reflex, are synchronized and coordinated by the intrinsic nervous network, extrinsic innervation, and pacemaker cells (Brookes et al., 2013; Smith and Koh, 2017; Fung and Vanden Berghe, 2020; Mercado-Perez and Beyder, 2022). Therefore, the vessel-enteric neuronal contacts might not exist and the systemic signals from the circulation may influence the enteric neurons *via* diffusion of the extracellular fluid.

In the mouse colon, we demonstrated nerve fibers immunoreactive for TH, CGRP, and PGP9.5 surrounding the blood vascular branches, but not the arterioles before the capillary networks in the submucosa layer. The innervation of the gut vessels is well-studied in humans and rodents (Li et al., 1998; De Fontgalland et al., 2008; Fu et al., 2013). In rodents, comprehensive data are mainly derived from guinea pigs and small intestines (Li et al., 1998; Fu et al., 2013). It is well-known that in the gut, the major contributor to mesenteric and submucosal blood vessel innervations is the sympathetic nervous system (Brookes et al., 2009; Lomax et al., 2010). TH nerves are mostly extrinsic, as demonstrated by the disappearance of TH immunolabeling after the extrinsic denervation of the ileum in guinea pigs (Li et al., 1998). Reports on the innervation of blood vessels in the human colon indicate that 85% contain TH and NPY, are rarely cholinergic, and few of the extrinsic afferents contain CGRP located around vessels (De Fontgalland et al., 2008). By contrast, in the mouse distal colon, anterograde tracing from the L6 and S1 dorsal root ganglia revealed that CGRP nerves projected to vessels (Spencer et al., 2014), and CGRP-ir nerve fibers run along the vessels in the mesentery (Peiris et al., 2017). In the muscularis externa and the capillary bed at the base of the mucosa crypts, immunolabeled nerves were rarely observed in the proximity of microvessels. In contrast, PGP9.5, CGRP, and VIP nerve terminals were closely distributed to the capillary net beneath the epithelia, providing neuroanatomical support that VIP and CGRP are neurotransmitters regulating mucosa secretion and blood flow (De Fontgalland et al., 2008; Mitsui et al., 2013; Fung and Vanden Berghe, 2020).

The enteric glia and macrophages are cells associated with vasculature involved in the communication between systemic signals and colonic functions. Cumulative evidence demonstrated that enteric glia are involved in gut inflammation and neurodegenerative disease (Cabarrocas et al., 2003; Rosenberg and Rao, 2021; Seguella and Gulbransen, 2021). However, the supportive morphological visualization of the glia-vascular contacts is not ascertained. In addition, it is notable that until recently, “many researchers failed to recognize the importance of differentiating the populations of enteric glia”. Sometimes the enteric glial cells are considered astroglia, the intestinal counterpart of the brain (Savidge et al., 2007; Gulbransen and Sharkey, 2012; Seguella and Gulbransen, 2021). In fact, as in the brain, enteric glial cells are heterogenic, labeled differently by the markers GFAP, S100, proteolipid protein 1, and Sox10 and classified as I–IV types by their morphology, location, and cellular markers (Gulbransen and Sharkey, 2012; Boesmans et al., 2015; Grundmann et al., 2019; Seguella and Gulbransen, 2021), and these molecules are not pan-expressed in the enteric glia (Benvenuti et al., 2020). While S100B and GFAP have been used interchangeably to identify glial cells in the intestine (Rosenberg and Rao, 2021), it is noted that the S100B antibody labels different types of enteric glia and other cells, while GFAP mostly labeled the glia in the enteric ganglia (Boesmans et al., 2015; Grundmann et al., 2019; Seguella and Gulbransen, 2021).

In the present study, we found that GFAP and S100B labeled various types of cells that were not frequently found in apposition to microvessels. In the mucosa and submucosa of the mouse proximal colon, there were only a few GFAP cells along the vessels. Likewise, in the enteric ganglia of mouse jejunum, a previous study using S100B and GFAP antibodies described “glial-endothelial contacts but not a continuous perivascular glial sheath or layer around the ganglia” (Fu et al., 2013). Other reports in the mouse small intestine also found that the GFAP-ir astrocyte-like cells were located at the top of the villi and their cell processes were close to the epithelium (Savidge et al., 2007). Although the morphologies of GFAP-ir glia are different in mouse and human colon (Grundmann et al., 2019), their locations are similar, mainly in the enteric plexus and intramuscular tissue, implying similar functions. In addition, the GFAP-containing enteric glia may be different from those in the central nervous system, which are isoforms and activated types of astrocytes (Middeldorp and Hol, 2011; Hol and Pekny, 2015; Liddelow and Barres, 2017). We found that the S100B-ir cell bodies and processes were distributed to the top of the mucosa but were rarely in adjacent apposition to the microvessels. Similarly, in the human colon, S100B-ir cells were densely distributed up to the top of the mucosa under the epithelium, and in the submucosa, perivascular S100B-ir cellular filaments were associated with CD34-labeled microvessels (Liu et al., 2013). The morphologies of the mucosal glial cells were different from those in the enteric nervous plexus, as mentioned in previous reports (Gulbransen and Sharkey, 2012; Boesmans et al., 2015; Seguella and Gulbransen, 2021). “A blood-enteric nervous system barrier” was proposed with ultrastructural evidence of an almost continuous layer of glial end-feet around the enteric ganglion (Gershon and Bursztajn, 1978; Sharkey, 2015; Dora et al., 2021). In the present study, we found little evidence for a possible contact between the glial cells and capillaries in the mouse colonic tissues. Collectively, these findings indicate that different types of glial cells and their location in colonic layers may be involved in various functions.

Microglia and macrophages both express Iba1 (Sasaki et al., 2001; Sasaki, 2017). Macrophages are heterogenic, tissue-specific, and change their morphology and phenotype under physiology and pathological status; therefore, various markers are expressed in macrophages (Mowat and Bain, 2011; Cipriani et al., 2016; Yip et al., 2021; Mischopoulou et al., 2022). Many of the markers recognize not only macrophages but also dendritic cells and other monocytes (Hume, 2008; Cipriani et al., 2016). Iba1 antibody can label resting, active, and phagocytic (amoeboid) macrophages/microglia and perivascular macrophages in mouse brains (Ravikumar et al., 2014; Sasaki, 2017). This marker has not been widely used for identifying colonic macrophages, especially in the mucosa. The Iba1-ir cells are considered resident macrophages in the intestine, and they are in the muscularis externa and submucosa (De Schepper et al., 2018a,b). However, Iba1 was also used as a marker to quantify the total population of macrophages in the mouse jejunum and their responsiveness to systemic lipopolysaccharide treatment (Mikkelsen et al., 2017), and to assess resident macrophages in the small intestine muscularis externa in response to inflammation in a postoperative ileus model (Enderes et al., 2020).

In the present study, Iba1-ir macrophages were found in all layers of mouse colonic segments and showed different morphology and relationships with microvessels. In the mucosa, the Iba1-ir macrophages were gathered close to microvessels and distributed densely along the characteristic anastomosed capillary rings labeled by WGA painting and CD31 immunoreactivity. Likewise, a recent study showed CX3CR1-labeled mucosa macrophages around CD31 immunostained blood vessels in the lamia propria of the colon and small intestine in mice (Honda et al., 2020). The presence of macrophages among the mucosal capillary networks shows possible direct communication with the circulation. In addition to blood providing a vital supply to the mucosa, the characteristic of this structural organization allowed the blood-immune-neural function, particularly with regard to cross-talk with the microbiome and immune system (Fung and Vanden Berghe, 2020). In the submucosa and muscularis externa, the Iba1-ir macrophages were not in a definite adjacent position to and not along the microvessels. This is consistent with a report in rat colon using markers CD163 and MHCII (Phillips and Powley, 2012). The anatomical location reflects the functional differentiation of macrophages in each layer (Honda et al., 2020). The macrophages were found to exert niche-specific functions in intestinal homeostasis, including the regulation of intestinal secretion and motility (Mikkelsen, 2010; Mowat and Bain, 2011; Muller et al., 2020; Mischopoulou et al., 2022). Macrophages confined to the muscularis externa have been shown to modulate peristalsis by a direct action on enteric neurons *via* the secretion of the growth factor bone morphogenetic protein 2 (BMP2) (Fung and Vanden Berghe, 2020; Ji et al., 2020; Mischopoulou et al., 2022). A role of macrophages in the submucosa has been reported to maintain the integrity of the submucosal vasculature (Bain and Schridde, 2018).

In summary, vascular painting by fluorescent tracers combined with immunofluorescent labeling highlighted features of vasculatures and their relationship to nerves, glia, and macrophages in the mouse colon, although our study did not explore the origins of the nerve fibers. There are segmental differences in the vascular formation and density between the proximal and distal colon, which is associated with tissue structures (e.g., mucosa folds and circular muscles in the proximal colon), but not with the vascular volume per tissue volume. The microvessels in the mucosa were morphologically different between the proximal and distal colon, namely, the capillary rings, individually in the crypts in the proximal colon and more than two in the distal colon, and the connecting microvessels between the top and bottom capillary net were more straight in the mid and distal colon than in the proximal colon. The continuous capillary rings near the lumen can be related to the absorption of water and nutrients, and also be involved in playing a role in the defense line, which is supported by the dense distribution of Iba1-positive macrophages around mucosal capillaries but random distribution in the muscle layers, with a few adjacent to vessels.

## Data availability statement

The raw data supporting the conclusions of this article will be made available by the authors, without undue reservation.

## Ethics statement

The animal study was reviewed and approved by Animal Research Committee at Veterans Affairs Greater Los Angeles Healthcare System.

## Author contributions

LW and YT initiated the studies and contributed to the conception. LW designed studies, conducted the experiments, acquired and segmented the images, analyzed the data, prepared the figures, and wrote the manuscript. YT edited the manuscript critically. P-QY participated in data interpretation and edited the manuscript. All authors contributed to the article and approved the submitted version.

## References

[B1] Alves C. H. Fernandes R. Santiago A. R. Ambrósio A. F. (2020). Microglia contribution to the regulation of the retinal and choroidal vasculature in age-related macular degeneration. Cells 9, 1217. 10.3390/cells9051217 32423062 PMC7290930

[B2] Araki K. Furuya Y. Kobayashi M. Matsuura K. Ogata T. Isozaki H. (1996). Comparison of mucosal microvasculature between the proximal and distal human colon. J. Electron. Microsc. 45, 202–206. 10.1093/oxfordjournals.jmicro.a023433 8765715

[B3] Bain C. C. Schridde A. (2018). Origin, differentiation, and function of intestinal macrophages. Front. Immunol. 9, 2733. 10.3389/fimmu.2018.02733 30538701 PMC6277706

[B4] Battistella R. Kritsilis M. Matuskova H. Haswell D. Cheng A. X. Meissner A.. (2021). Not all lectins are equally suitable for labeling rodent vasculature. Int. J. Mol. Sci. 22, 11554. 10.3390/ijms222111554 34768985 PMC8584019

[B5] Bennett H. C. Kim Y. (2022). Advances in studying whole mouse brain vasculature using high-resolution 3D light microscopy imaging. Neurophotonics 9, 021902. 10.1117/1.NPh.9.2.021902 35402638 PMC8983067

[B6] Benvenuti L. D'Antongiovanni V. Pellegrini C. Antonioli L. Bernardini N. Blandizzi C.. (2020). Enteric glia at the crossroads between intestinal immune system and epithelial barrier: implications for parkinson disease. Int. J. Mol. Sci. 21, 9199. 10.3390/ijms21239199 33276665 PMC7730281

[B7] Boesmans W. Lasrado R. Vanden Berghe P. Pachnis V. (2015). Heterogeneity and phenotypic plasticity of glial cells in the mammalian enteric nervous system. Glia 63, 229–241. 10.1002/glia.22746 25161129

[B8] Boley S. J. Sammartano R. Adams A. DiBiase A. Kleinhaus S. Sprayregen S. (1977). On the nature and etiology of vascular ectasias of the colon. Degenerative lesions of aging. Gastroenterology 72(4 Pt 1), 650–660. 10.1016/S0016-5085(77)80149-2 300063

[B9] Brookes S. J. Dinning P. G. Gladman M. A. (2009). Neuroanatomy and physiology of colorectal function and defaecation: from basic science to human clinical studies. Neurogastroenterol. Motil. 21(Suppl. 2), 9–19. 10.1111/j.1365-2982.2009.01400.x 19824934

[B10] Brookes S. J. Spencer N. J. Costa M. Zagorodnyuk V. P. (2013). Extrinsic primary afferent signalling in the gut. Nat. Rev. Gastroenterol. Hepatol. 10, 286–296. 10.1038/nrgastro.2013.29 23438947

[B11] Browning J. Gannon B. (1986). Mucosal microvascular organization of the rat colon. Acta Anat. 126, 73–77. 10.1159/000146191 3739612

[B12] Cabarrocas J. Savidge T. C. Liblau R. S. (2003). Role of enteric glial cells in inflammatory bowel disease. Glia 41, 81–93. 10.1002/glia.10169 12465048

[B13] Cao Y. Wang H. Wang Q. Han X. Zeng W. (2018). Three-dimensional volume fluorescence-imaging of vascular plasticity in adipose tissues. Mol. Metab 14, 71–81. 10.1016/j.molmet.2018.06.004 29914852 PMC6034070

[B14] Chang Y. S. di Tomaso E. McDonald D. M. Jones R. Jain R. K. Munn L. L. (2000). Mosaic blood vessels in tumors: frequency of cancer cells in contact with flowing blood. Proc. Natl. Acad. Sci. U. S. A. 97, 14608–14613. 10.1073/pnas.97.26.14608 11121063 PMC18966

[B15] Cipriani G. Gibbons S. J. Kashyap P. C. Farrugia G. (2016). Intrinsic gastrointestinal macrophages: their phenotype and role in gastrointestinal motility. Cell Mol. Gastroenterol. Hepatol. 2, 120–130. 10.1016/j.jcmgh.2016.01.003 27047989 PMC4817106

[B16] Cirillo C. Sarnelli G. Esposito G. Turco F. Steardo L. Cuomo R. (2011). S100B protein in the gut: the evidence for enteroglial-sustained intestinal inflammation. World J. Gastroenterol. 17, 1261–1266. 10.3748/wjg.v17.i10.1261 21455324 PMC3068260

[B17] Clairembault T. Leclair-Visonneau L. Neunlist M. Derkinderen P. (2015). Enteric glial cells: new players in Parkinson's disease? Mov. Disord. 30, 494–498. 10.1002/mds.25979 25100667

[B18] de Fontgalland D. Brookes S. J. Gibbins I. Sia T. C. Wattchow D. A. (2014). The neurochemical changes in the innervation of human colonic mesenteric and submucosal blood vessels in ulcerative colitis and Crohn's disease. Neurogastroenterol. Motil. 26, 731–744. 10.1111/nmo.12327 24597665

[B19] De Fontgalland D. Wattchow D. A. Costa M. Brookes S. J. (2008). Immunohistochemical characterization of the innervation of human colonic mesenteric and submucosal blood vessels. Neurogastroenterol. Motil. 20, 1212–1226. 10.1111/j.1365-2982.2008.01150.x 18643894

[B20] De Schepper S. Stakenborg N. Matteoli G. Verheijden S. Boeckxstaens G. E. (2018a). Muscularis macrophages: key players in intestinal homeostasis and disease. Cell. Immunol. 330, 142–150. 10.1016/j.cellimm.2017.12.009 29291892 PMC6108422

[B21] De Schepper S. Verheijden S. Aguilera-Lizarraga J. Viola M. F. Boesmans W. Stakenborg N.. (2018b). Self-maintaining gut macrophages are essential for intestinal homeostasis. Cell 175, 400–415. 10.1016/j.cell.2018.07.048 30173915

[B22] den Uil S. H. van den Broek E. Coupé V. M. H. Vellinga T. T. Delis-van Diemen P. M. Bril H.. (2019). Prognostic value of microvessel density in stage II and III colon cancer patients: a retrospective cohort study. BMC Gastroenterol. 19, 146. 10.1186/s12876-019-1063-4 31420015 PMC6698008

[B23] Dora D. Ferenczi S. Stavely R. Toth V. E. Varga Z. V. Kovacs T.. (2021). Evidence of a myenteric plexus barrier and its macrophage-dependent degradation during murine colitis: implications in enteric neuroinflammation. Cell Mol. Gastroenterol. Hepatol. 12, 1617–1641. 10.1016/j.jcmgh.2021.07.003 34246810 PMC8551790

[B24] Enderes J. Mallesh S. Schneider R. Hupa K. J. Lysson M. Schneiker B.. (2020). A population of radio-resistant macrophages in the deep myenteric plexus contributes to postoperative ileus via toll-like receptor 3 signaling. Front. Immunol. 11, 581111. 10.3389/fimmu.2020.581111 33519804 PMC7838642

[B25] Fu Y. Y. Peng S. J. Lin H. Y. Pasricha P. J. Tang S. C. (2013). 3-D imaging and illustration of mouse intestinal neurovascular complex. Am. J. Physiol. Gastrointest. Liver Physiol. 304, G1–11. 10.1152/ajpgi.00209.2012 23086917

[B26] Fung C. Vanden Berghe P. (2020). Functional circuits and signal processing in the enteric nervous system. Cell. Mol. Life Sci. 77, 4505–4522. 10.1007/s00018-020-03543-6 32424438 PMC7599184

[B27] Gabella G. (1972). Fine structure of the myenteric plexus in the guinea-pig ileum. J. Anat. 111 (Pt 1), 69–97. 4335909 PMC1271115

[B28] Gershon M. D. Bursztajn S. (1978). Properties of the enteric nervous system: limitation of access of intravascular macromolecules to the myenteric plexus and muscularis externa. J. Comp. Neurol. 180, 467–488. 10.1002/cne.901800305 659670

[B29] Gibert-Ramos A. Sanfeliu-Redondo D. Aristu-Zabalza P. Martínez-Alcocer A. Gracia-Sancho J. Guixé-Muntet S.. (2021). The hepatic sinusoid in chronic liver disease: the optimal milieu for cancer. Cancers 13, 5719. 10.3390/cancers13225719 34830874 PMC8616349

[B30] Grundmann D. Loris E. Maas-Omlor S. Huang W. Scheller A. Kirchhoff F.. (2019). Enteric glia: S100, GFAP, and beyond. Anat. Rec. 302, 1333–1344. 10.1002/ar.24128 30951262

[B31] Gulbransen B. D. Sharkey K. A. (2012). Novel functional roles for enteric glia in the gastrointestinal tract. Nat. Rev. Gastroenterol. Hepatol. 9, 625–632. 10.1038/nrgastro.2012.138 22890111

[B32] Hill G. S. Heudes D. Bariéty J. (2003). Morphometric study of arterioles and glomeruli in the aging kidney suggests focal loss of autoregulation. Kidney Int. 63, 1027–1036. 10.1046/j.1523-1755.2003.00831.x 12631084

[B33] Hol E. M. Pekny M. (2015). Glial fibrillary acidic protein (GFAP) and the astrocyte intermediate filament system in diseases of the central nervous system. Curr. Opin. Cell Biol. 32, 121–130. 10.1016/j.ceb.2015.02.004 25726916

[B34] Honda M. Surewaard B. G. J. Watanabe M. Hedrick C. C. Lee W. Y. Brown K.. (2020). Perivascular localization of macrophages in the intestinal mucosa is regulated by Nr4a1 and the microbiome. Nat. Commun. 11, 1329. 10.1038/s41467-020-15068-4 32165624 PMC7067862

[B35] Hugenholtz F. de Vos W. M. (2018). Mouse models for human intestinal microbiota research: a critical evaluation. Cell. Mol. Life Sci. 75, 149–160. 10.1007/s00018-017-2693-8 29124307 PMC5752736

[B36] Hume D. A. (2008). Macrophages as APC and the dendritic cell myth. J. Immunol. 181, 5829–5835. 10.4049/jimmunol.181.9.5829 18941170

[B37] Jaffey D. M. Chesney L. Powley T. L. (2021). Stomach serosal arteries distinguish gastric regions of the rat. J. Anat. 239, 903–912. 10.1111/joa.13480 34142374 PMC8450471

[B38] Ji S. Traini C. Mischopoulou M. Gibbons S. J. Ligresti G. Faussone-Pellegrini M. S.. (2020). Muscularis macrophages establish cell-to-cell contacts with telocytes/PDGFRα-positive cells and smooth muscle cells in the human and mouse gastrointestinal tract. Neurogastroenterol. Motil. 7, e13993. 10.1111/nmo.13993 33020982 PMC7902307

[B39] Kampoli K. Foukas P. G. Ntavatzikos A. Arkadopoulos N. Koumarianou A. (2022). Interrogating the interplay of angiogenesis and immunity in metastatic colorectal cancer. World J. Methodol. 12, 43–53. 10.5662/wjm.v12.i1.43 35117981 PMC8790311

[B40] Király A. Süt,ö G. Guth P. H. Tach,é Y. (1998). Peripheral mediators involved in gastric hyperemia to vagal activation by central TRH analog in rats. Am. J. Physiol. 274, G170–177. 10.1152/ajpgi.1998.274.1.G170 9458786

[B41] Konerding M. A. Fait E. Gaumann A. (2001). 3D microvascular architecture of pre-cancerous lesions and invasive carcinomas of the colon. Br. J. Cancer 84, 1354–1362. 10.1054/bjoc.2001.1809 11355947 PMC2363651

[B42] Korotinski S. Katz A. Malnick S. D. (2005). Chronic ischaemic bowel diseases in the aged–go with the flow. Age Ageing 34, 10–16. 10.1093/ageing/afh226 15591479

[B43] Li Y. Song Y. Zhao L. Gaidosh G. Laties A. M. Wen R. (2008). Direct labeling and visualization of blood vessels with lipophilic carbocyanine dye DiI. Nat. Protoc. 3, 1703–1708. 10.1038/nprot.2008.172 18846097 PMC2811090

[B44] Li Z. S. Fox-Threlkeld J. E. Furness J. B. (1998). Innervation of intestinal arteries by axons with immunoreactivity for the vesicular acetylcholine transporter (VAChT). J. Anat. 192 (Pt 1), 107–117. 10.1046/j.1469-7580.1998.19210107.x 9568566 PMC1467744

[B45] Liddelow S. A. Barres B. A. (2017). Reactive astrocytes: production, function, and therapeutic potential. Immunity 46, 957–967. 10.1016/j.immuni.2017.06.006 28636962

[B46] Liu Y. A. Chung Y. C. Pan S. T. Shen M. Y. Hou Y. C. Peng S. J.. (2013). 3-D imaging, illustration, and quantitation of enteric glial network in transparent human colon mucosa. Neurogastroenterol. Motil. 25, e324–338. 10.1111/nmo.12115 23495930

[B47] Lomax A. E. Sharkey K. A. Furness J. B. (2010). The participation of the sympathetic innervation of the gastrointestinal tract in disease states. Neurogastroenterol. Motil. 22, 7–18. 10.1111/j.1365-2982.2009.01381.x 19686308

[B48] Luxán G. Dimmeler S. (2022). The vasculature: a therapeutic target in heart failure? Cardiovasc. Res. 118, 53–64. 10.1093/cvr/cvab047 33620071 PMC8752358

[B49] Ma B. von Wasielewski R. Lindenmaier W. Dittmar K. E. (2007). Immmunohistochemical study of the blood and lymphatic vasculature and the innervation of mouse gut and gut-associated lymphoid tissue. Anat. Histol. Embryol. 36, 62–74. 10.1111/j.1439-0264.2006.00741.x 17266671

[B50] Mercado-Perez A. Beyder A. (2022). Gut feelings: mechanosensing in the gastrointestinal tract. Nat. Rev. Gastroenterol. Hepatol. 19, 283–296. 10.1038/s41575-021-00561-y 35022607 PMC9059832

[B51] Mestres P. Diener M. Rummel W. (1992). Electron microscopy of the mucosal plexus of the rat colon. Acta Anat. 143, 275–282. 10.1159/000147262 1502867

[B52] Middeldorp J. Hol E. M. (2011). GFAP in health and disease. Prog. Neurobiol. 93, 421–443. 10.1016/j.pneurobio.2011.01.005 21219963

[B53] Mikkelsen H. B. (2010). Interstitial cells of Cajal, macrophages and mast cells in the gut musculature: morphology, distribution, spatial and possible functional interactions. J. Cell. Mol. Med. 14, 818–832. 10.1111/j.1582-4934.2010.01025.x 20132411 PMC3823114

[B54] Mikkelsen H. B. Huizinga J. D. Larsen J. O. Kirkeby S. (2017). Ionized calcium-binding adaptor molecule 1 positive macrophages and HO-1 up-regulation in intestinal muscularis resident macrophages. Anat. Rec. 300, 1114–1122. 10.1002/ar.23517 27860408 PMC5484384

[B55] Mischopoulou M. D'Ambrosio M. Bigagli E. Luceri C. Farrugia G. Cipriani G. (2022). Role of macrophages and mast cells as key players in the maintenance of gastrointestinal smooth muscle homeostasis and disease. Cell Mol. Gastroenterol. Hepatol. 13, 1849–1862. 10.1016/j.jcmgh.2022.02.017 35245688 PMC9123576

[B56] Mitsui R. Miyamoto S. Takano H. Hashitani H. (2013). Properties of submucosal venules in the rat distal colon. Br. J. Pharmacol. 170, 968–977. 10.1111/bph.12347 23992146 PMC3949646

[B57] Mowat A. M. Bain C. C. (2011). Mucosal macrophages in intestinal homeostasis and inflammation. J. Innate Immun. 3, 550–564. 10.1159/000329099 22025201 PMC3224516

[B58] Mühlfeld C. (2021). Stereology and three-dimensional reconstructions to analyze the pulmonary vasculature. Histochem. Cell Biol. 156, 83–93. 10.1007/s00418-021-02013-9 34272602 PMC8397636

[B59] Müller A. M. Hermanns M. I. Skrzynski C. Nesslinger M. Müller K. M. Kirkpatrick C. J. (2002). Expression of the endothelial markers PECAM-1, vWf, and CD34 *in vivo* and *in vitro*. Exp. Mol. Pathol. 72, 221–229. 10.1006/exmp.2002.2424 12009786

[B60] Muller P. A. Matheis F. Mucida D. (2020). Gut macrophages: key players in intestinal immunity and tissue physiology. Curr. Opin. Immunol. 62, 54–61. 10.1016/j.coi.2019.11.011 31841704 PMC7067652

[B61] Murray I. Paolini M. A. (2022). Histology, Kidney and Glomerulus. Treasure Island, FL: StatPearls Publishing. 32119431

[B62] Nava G. M. Friedrichsen H. J. Stappenbeck T. S. (2011). Spatial organization of intestinal microbiota in the mouse ascending colon. ISME J. 5, 627–638. 10.1038/ismej.2010.161 20981114 PMC3105732

[B63] Peiris M. Hockley J. R. Reed D. E. Smith E. S. J. Bulmer D. C. Blackshaw L. A. (2017). Peripheral K(V)7 channels regulate visceral sensory function in mouse and human colon. Mol. Pain 13, 1744806917709371. 10.1177/1744806917709371 28566000 PMC5456027

[B64] Pénicaud L. (2017). Autonomic nervous system and pancreatic islet blood flow. Biochimie 143, 29–32. 10.1016/j.biochi.2017.10.001 29017926

[B65] Phillips R. J. Powley T. L. (2012). Macrophages associated with the intrinsic and extrinsic autonomic innervation of the rat gastrointestinal tract. Auton. Neurosci. 169, 12–27. 10.1016/j.autneu.2012.02.004 22436622 PMC3361649

[B66] Porvasnik S. L. Mah C. Polyak S. (2010). Targeting murine small bowel and colon through selective superior mesenteric artery injection. Microsurgery 30, 487–493. 10.1002/micr.20767 20238384

[B67] Ravikumar M. Sunil S. Black J. Barkauskas D. S. Haung A. Y. Miller R. H.. (2014). The roles of blood-derived macrophages and resident microglia in the neuroinflammatory response to implanted intracortical microelectrodes. Biomaterials 35, 8049–8064. 10.1016/j.biomaterials.2014.05.084 24973296 PMC4169074

[B68] Ravnic D. J. Jiang X. Wolloscheck T. Pratt J. P. Huss H. Mentzer S. J.. (2005). Vessel painting of the microcirculation using fluorescent lipophilic tracers. Microvasc. Res. 70, 90–96. 10.1016/j.mvr.2005.06.002 16095629

[B69] Ravnic D. J. Konerding M. A. Tsuda A. Huss H. T. Wolloscheck T. Pratt J. P.. (2007). Structural adaptations in the murine colon microcirculation associated with hapten-induced inflammation. Gut 56, 518–523. 10.1136/gut.2006.101824 17114297 PMC1856840

[B70] Rosenberg H. J. Rao M. (2021). Enteric glia in homeostasis and disease: from fundamental biology to human pathology. iScience 24, 102863. 10.1016/j.isci.2021.102863 34401661 PMC8348155

[B71] Sasaki A. (2017). Microglia and brain macrophages: an update. Neuropathology 37, 452–464. 10.1111/neup.12354 27859676

[B72] Sasaki Y. Ohsawa K. Kanazawa H. Kohsaka S. Imai Y. (2001). Iba1 is an actin-cross-linking protein in macrophages/microglia. Biochem. Biophys. Res. Commun. 286, 292–297. 10.1006/bbrc.2001.5388 11500035

[B73] Savidge T. C. Sofroniew M. V. Neunlist M. (2007). Starring roles for astroglia in barrier pathologies of gut and brain. Lab. Invest. 87, 731–736. 10.1038/labinvest.3700600 17607301

[B74] Scalise A. A. Kakogiannos N. Zanardi F. Iannelli F. Giannotta M. (2021). The blood-brain and gut-vascular barriers: from the perspective of claudins. Tissue Barr. 9, 1926190. 10.1080/21688370.2021.1926190 34152937 PMC8489939

[B75] Schneider S. Wright C. M. Heuckeroth R. O. (2019). Unexpected roles for the second brain: enteric nervous system as master regulator of bowel function. Annu. Rev. Physiol. 81, 235–259. 10.1146/annurev-physiol-021317-121515 30379617

[B76] Seguella L. Gulbransen B. D. (2021). Enteric glial biology, intercellular signalling and roles in gastrointestinal disease. Nat. Rev. Gastroenterol. Hepatol. 18, 571–587. 10.1038/s41575-021-00423-7 33731961 PMC8324524

[B77] Sharkey K. A. (2015). Emerging roles for enteric glia in gastrointestinal disorders. J. Clin. Invest. 125, 918–925. 10.1172/JCI76303 25689252 PMC4362226

[B78] Skinner S. A. O'Brien P. E. (1996). The microvascular structure of the normal colon in rats and humans. J. Surg. Res. 61, 482–490. 10.1006/jsre.1996.0151 8656630

[B79] Smith T. K. Koh S. D. (2017). A model of the enteric neural circuitry underlying the generation of rhythmic motor patterns in the colon: the role of serotonin. Am. J. Physiol. Gastrointest. Liver Physiol. 312, G1–G14. 10.1152/ajpgi.00337.2016 27789457 PMC5283906

[B80] Spencer N. J. Kyloh M. Duffield M. (2014). Identification of different types of spinal afferent nerve endings that encode noxious and innocuous stimuli in the large intestine using a novel anterograde tracing technique. PLoS ONE 9, e112466. 10.1371/journal.pone.0112466 25383884 PMC4226564

[B81] Sun X. F. Zhang H. Wu X. C. Han Y. M. Hou G. Q. Xian M. S. (1992). Microvascular corrosion casting of normal tissue, transitional mucosa and adenocarcinoma in the human colon. Acta Oncol. 31, 37–40. 10.3109/02841869209088262 1586502

[B82] Tsai P. S. Kaufhold J. P. Blinder P. Friedman B. Drew P. J. Karten H. J.. (2009). Correlations of neuronal and microvascular densities in murine cortex revealed by direct counting and colocalization of nuclei and vessels. J. Neurosci. 29, 14553–14570. 10.1523/JNEUROSCI.3287-09.2009 19923289 PMC4972024

[B83] Ungvari Z. Tarantini S. Kiss T. Wren J. D. Giles C. B. Griffin C. T.. (2018). Endothelial dysfunction and angiogenesis impairment in the ageing vasculature. Nat. Rev. Cardiol. 15, 555–565. 10.1038/s41569-018-0030-z 29795441 PMC6612360

[B84] Wang L. Yuan P. Q. Challis C. Ravindra Kumar S. Taché Y. (2022). Transduction of systemically administered adeno-associated virus in the colonic enteric nervous system and c-kit cells of adult mice. Front. Neuroanat. 16, 884280. 10.3389/fnana.2022.884280 35734536 PMC9207206

[B85] Yang B. Treweek J. B. Kulkarni R. P. Deverman B. E. Chen C. K. Lubeck E.. (2014). Single-cell phenotyping within transparent intact tissue through whole-body clearing. Cell 158, 945–958. 10.1016/j.cell.2014.07.017 25088144 PMC4153367

[B86] Yip J. L. K. Balasuriya G. K. Spencer S. J. Hill-Yardin E. L. (2021). The role of intestinal macrophages in gastrointestinal homeostasis: heterogeneity and implications in disease. Cell Mol. Gastroenterol. Hepatol. 12, 1701–1718. 10.1016/j.jcmgh.2021.08.021 34506953 PMC8551786

[B87] Yuan P. Q. Bellier J. P. Li T. Kwaan M. R. Kimura H. Tach,é Y. (2021). Intrinsic cholinergic innervation in the human sigmoid colon revealed using CLARITY, three-dimensional (3D) imaging, and a novel anti-human peripheral choline acetyltransferase (hpChAT) antiserum. Neurogastroenterol. Motil. 33, e14030. 10.1111/nmo.14030 33174295 PMC8126258

[B88] Zhu C. Rajendran P. S. Hanna P. Efimov I. R. Salama G. Fowlkes C. C.. (2022). High-resolution structure-function mapping of intact hearts reveals altered sympathetic control of infarct border zones. JCI Insight 7, e153913. 10.1172/jci.insight.153913 35132963 PMC8855798

